# New Terpenoids from *Viguiera dentata*: In Silico Pesticide‐Likeness Properties, Acetylcholinesterase Inhibition, Molecular Docking, and Evaluation against *Spodoptera frugiperda*

**DOI:** 10.1002/cbdv.202500917

**Published:** 2025-05-06

**Authors:** Amira Arciniegas, Olivia Pérez‐Valera, Simón Hernández‐Ortega, Antonio Nieto‐Camacho, Israel Valencia, Joel Daniel Castañeda‐Espinoza, Rodolfo Figueroa‐Brito, José Luis Villaseñor, Guillermo Delgado

**Affiliations:** ^1^ Instituto de Química Universidad Nacional Autónoma de México, Circuito Exterior, Ciudad Universitaria, Ciudad de México; ^2^ Laboratorio de Farmacobiología Universidad Nacional Autónoma de México, FES Iztacala, Tlalnepantla de Baz, México; ^3^ Centro de Desarrollo de Productos Bióticos (CEPROBI) Instituto Politécnico Nacional, Yautepec, México; ^4^ Instituto de Biología Universidad Nacional Autónoma de México, Circuito Exterior, Ciudad Universitaria, Ciudad de México

**Keywords:** acetylcholinesterase, in silico pesticide‐like properties, insecticidal activity, natural products, terpenes

## Abstract

The chemical analysis of the melliferous plant *Viguiera dentata* (Asteraceae) yielded cycloartanes **1–9** (including the new compounds **7–9**), *ent*‐kaurenes (**10–15**), diversifolin (**16)** and other constituents. The structure of **9** was confirmed by X‐ray analysis. To evaluate the insecticidal potential of its constituents, in silico pesticide‐likeness calculations for structures **1–16** were performed indicating no violations of the Tice rules. Tests for activity against acetylcholinesterase revealed that only cycloartanes **1** (78.85 µM) and **6** (53.54 µM) inhibited the enzyme. Molecular docking analysis showed interactions between compounds **1**, **10**, and **13**, with Y337, a key amino acid in the catalytic site. A bioassay against *Spodoptera frugiperda* revealed that compounds **1**, **2**, **9**, **10**, and **13** displayed activity (50% lethal concentration for larval mortality [LC_50_] 51.61, 84.56, 99.66, 24.69, and 62.40 ppm, respectively; reference: betulinic acid LC_50_ 94.25 ppm). Thus, specific cycloartanes and *ent*‐kaurenes were identified as insecticidal compounds of *V. dentata* against *S. frugiperda*.

## Graphical abstract

The chemical analysis of aerial parts of the melliferous *Viguiera dentata* led to the isolation of 20 substances, including the new cycloartanes 24‐*epi*‐argentatin C, 7β‐hydroxy‐24‐*epi*‐argentatin C, 7β‐hydroxy‐argentatin B. In‐combo results indicated that specific argentatins and *ent*‐kaurenoic acids have the potential to control *Spodoptera frugiperda*

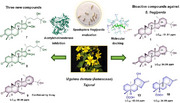

## 1 Introduction

*Viguiera* (Asteraceae, Heliantheae, Helianthinae) is an American genus initially described by Blake in 1918, with 141 species [1], and revised by Schilling and Panero in 2011 [2] to have most of its species segregated into ten genera [3]. This last classification limited the genus to nine species [4]. Previous chemical studies on the melliferous *V. dentata* (common name: tajonal) have led to the isolation mainly of *ent*‐kaurenoids and cycloartane derivatives, together with diversifolin, spathulenol, and manool [5, 6, 7, 8]. The composition of its essential oil and biological activities have also been described [9], including the volatile components of the nectariferous flowers, whose honey is much appreciated [10, 11]. The present study describes the chemical composition of a population of *V. dentata* (Cav.) Spreng. Nine cycloartane derivatives (**1–9**), including the previously unreported (**7–9**), six *ent‐*kauranes (**10–15**), one sesquiterpene lactone (**16**), verbenol, caryophyllene oxide, and two phytosterols, were isolated. Cycloartane‐type compounds exhibit a wide range of pharmacological properties, including anti‐tumor, anti‐osteoporosis, anti‐VIH, anti‐parasite, and anti‐tuberculosis activities [12], as well as insecticidal properties [13]. Similarly, *ent*‐kaurane derivatives possess anti‐inflammatory, anti‐tumor, anti‐bacterial [14], insecticidal, and antifeedant properties [15].

In silico evaluation of the pesticide‐likeness properties of natural products is a valuable tool to assess their biological potential [16], and most cycloartanes and *ent*‐kauranes isolated from *V. dentata* exhibited pesticide potential, as they did not violate the Tice rules. Even though this enzyme (acetylcholinesterase [AChE]) is a major target in the development of new insecticides [17], only a few studies have examined its AChE inhibitory activities. Previous docking studies have indicated that some *ent*‐kauranes have insecticidal effects by inhibiting AChE [18]. AChE inhibitors bind to the enzyme and interfere with the breakdown of acetylcholine, disrupting neurotransmission [17]. None of the isolated compounds has been tested as AChE inhibitors, except *ent*‐kaurenoic acid (**10**) [19, 20]. The AChE inhibitory activity of the isolated compounds was evaluated, and their interaction with the enzyme was determined using molecular docking techniques. Additionally, four cycloartanes (**1**, **2**, **6**, and **9**) and two *ent‐*kaurene acids (**10** and **13**) were tested for insecticidal activity against *Spodoptera frugiperda*, a major maize pest.

## 2 Results and Discussion

**FIGURE 1:**
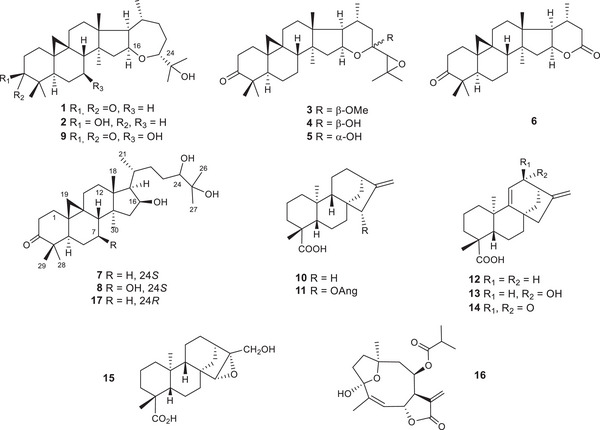
Chemical structures of compounds **1**–**17**.

Chemical analysis of *V. dentata* led to the identification of argentatin B (**1**) [21, 22], argentatin D (**2**) [23], cycloartanones (**3–6**) [5], 24‐*epi*‐argentatin C (**7**), 7β‐hydroxy‐24‐*epi*‐argentatin C (**8**), 7β‐hydroxyargentatin B (**9**), *ent*‐kaurenoic acid (**10**) [24], angeloylgrandifloric acid (**11**) [25], grandiflorenic acid (**12**) [21], 12α‐hydroxy*‐ent*‐kaur‐9(11),16‐dien‐19‐oic acid (**13**) [26], *ent*‐12‐oxo‐kaura‐9(11),16‐dien‐19‐oic acid (**14**) [27], 15α,16α‐epoxy‐*ent*‐17‐hydroxykauran‐19‐oic‐acid (**15**) [28], and diversifolin (**16**) [6] (Figure 1). Additionally, β‐sitosterol, stigmasterol, verbenol, and caryophyllene oxide were characterized. The structures of these compounds were elucidated using spectroscopic and spectrometric methods, with known compounds compared to published data and authentic samples.

### 2.1 Structural Identification of New Compounds

Compound **7** had the molecular formula C_30_H_50_O_4_ determined from high‐resolution direct analysis in real‐time mass spectrometry (HRDARTMS) and carbon‐13 nuclear magnetic resonance (^13^C NMR). Its infrared (IR) spectrum exhibited absorption bands for hydroxy (3402 cm^−1^) and carbonyl (1701 cm^−1^) groups. The NMR spectra (Tables 1 and 2) were similar to those of known compounds **1–5**, featuring six tertiary and one secondary methyl group, and the characteristic protons of a tetra‐substituted cyclopropyl methylene group, indicating a cycloartane skeleton. The proton NMR (^1^H NMR) spectrum exhibited the resonances of two oxygenated methine protons at δ_H_ 4.49 (dt 5.1, 7.8 Hz) and δ_H_ 3.39 (dd 10.5, 1.8 Hz), assigned to H‐16 and H‐24, respectively, by their correlation spectroscopy (COSY) and heteronuclear multiple bond correlations (HMBCs). The ^13^C NMR spectrum exhibited 30 carbon atoms, including a carbonyl group, the oxymethine carbons C‐16 (δ_C_ 72.7) and C‐24 (δ_C_ 80.4), and a tertiary carbon‐bearing oxygen atom at C‐25 (δ_C_ 73.0). HMBC correlations of CH_3_‐28 (δ_H_ 1.06 s), CH_3_‐29 (δ_H_ 1.11 s), H_2_‐2 (δ_H_ 2.72, td 13.8, 6.3 Hz, H‐2β and 2.31, brd 13.8, H‐2α), and H_2_‐1 (δ_H_ 1.86 m, H‐1α and 1.56 m, H‐1β) with the carbonyl group at δ_C_ 216.6 defined as a ketone group at C‐3. Compound **7** had the same substitution pattern as argentatin C (**17**) [23], and an identical CD spectrum, with a negative Cotton effect at λ 299 nm. However, its melting point, optical rotation, and thin layer chromatography (TLC) retention factor differed from those of authentic sample **17** [23]. The resonances of the side chain atoms C‐20 to C‐24 shifted downfield Δδ by 4.9, 0.7, 2.7, 2.4, and 5.2 ppm, respectively, compared to those of **17** (Table 2). Since the connectivity and stereochemistry of the cycloartane moiety in these compounds were identical, compound **7** should be the 24*S* epimer of argentatin C. These results are similar to reports of several 24*S*,25‐dihydroxycycloartane derivatives [29, 30]. Spectroscopic data for compound **17** are provided in Tables 1 and 2, as they are not available in the literature.

**TABLE 1:** Proton nuclear magnetic resonance (^1^H NMR) spectroscopic data (CDCl_3_) for compounds **7**–**9** and **17** (δH, J in Hz).

Position	7^[a]^	8^[^ ^b^, ^d^ ^]^	9^[^ ^c^, ^d^ ^]^	17^[a]^
1^a^	1.86, m	1.82, m	1.82, td (13.6, 4.4)	1.87, m
1b	1.56, m	1,65, m	1.65, m	1.58, m
2β	2.72, td (13.8, 6.3)	2.78, td (14.0, 6.3)	2.76, td (14.0, 6.8)	2.71, td (13.8, 6.3)
2α	2.31, brd (13.8)	2.27, dt (14.0, 2.1)	2.30, ddd (14.0, 4.4, 2.8)	2.31, ddd (13.8, 4.2, 3.0)
5α	1.72, m	1.93, dd (13.3, 4.2)	1.91, dd (13.2, 4.0)	1.71, m
6α	1.61, m	1.71, m	1.68, m	1.63, m
6β	1.17–1.11, m	1.18, m	1.20, m	1.17–1.11, m
7α	1.40, m	3.56 ddd (10.5, 9.1, 3.5)	3.62, ddd (12.6, 9.2, 4.0)	1.0–1.3, m
7b	1.17–1.11, m			1.17‐1.11, m
8β	1.70, m	1.78, d (9.1)	1.76, d (9.2)	1.70, m
11a	2.04, m	1.98, ddd (15.4, 9.8, 5.6)	1.94, m	2.09, m
11b	1.17–1.11, m	1.36, ddd (15.4, 10.5, 4.9)	1.37, m	1.2‐1.1, m
12	1.67, m	1.82, m	1.69, m	1.68, m
15α	2.03, dd (13.2, 7.8)	2.26, dd (14.0, 5.6)	2.13, m	2.07, m
15β	1.39, dd 13.2, 5.1)	1.67, m	1.68, m	1.37, m
16	4.49, dt (5.1, 7.8)	4.42, dt (5.6, 8.4)	4.65, q (7.6)	4.50, dt (5.1, 7.8)
17	1.70, m	1.65, m	1.59, m	1.70, m
18	1.20, s	1.24, s	1.20, s	1.20, s
19a	0.82, d (4.2)	0.89, d (4.9)	under CH_3_ 21	0.83, d (4.5)
19b	0.59, d (4.2)	0.66, d (4.9)	0.60, d (4.4)	0.60, d (4.5)
20	1.85, m	1.85, m	2.08, m	1.91, m
21	0.96, d (6.0)	1.00, d (7.0)	0.95, d (6.4)	0.95, d (6.6)
22a	1.81, m	1.93, m	1.70, m	1.74, m
22b	1.17–1.11, m	1.05, m	1.40, m	1.60, m
23	1.70, m	1.70, m	2.03, m	1.61, m
23b	1.32, m	1.21, m	1.62, m	1.41, m
24	3.39, dd (10.5, 1.8)	3.26, dd (11.2, 2.1)	3.53, dd (12.4, 2.0)	3.59, dd (11.3, 2.7)
26	1.17, s	1.12, s	1.13, s	1.26, s
27	1.22, s	1.16, s	1.08, s	1.17, s
28	1.06, s	1.06, s	1.03, s	1.06, s
29	1.11, s	1.14, s	1.11, s	1.11, s
30	0.91, s	0.98, s	0.95, s	0.91, s

^[a]^ 300 MHz.

^[b]^ 700 MHz.

^[c]^ 400 MHz.

^[d]^ CD_3_OD.

**TABLE 2:** Carbon‐13 nuclear magnetic resonance (^13^C NMR) spectroscopic data (CDCl_3_) for compounds **7**–**9** and **17**.

Position, type	7^[a]^	8 ^[^ ^b^, ^d^ ^]^	9 ^[^ ^c^, ^d^ ^]^	17 ^[a]^
1, CH_2_	33.4	32.5	32.4	33.4
2, CH_2_	37.4	36.7	36.7	37.4
3, C	216.6	217.0	216.9	216.4
4, C	50.2	49.5	49.4	50.2
5, CH	48.4	47.0	46.8	48.4
6, CH_2_	21.4	30.9	30.8	21.4
7, CH_2_	25.9	69.7*^e^*	69.5^[e]^	25.9
8, CH	47.8	54.2	53.6	47.9
9, C	20.9		20.6	20.9
10, C	26.0	26.4	26.3	26.1
11, CH_2_	26.4	26.3	26.2	26.4
12, CH_2_	32.5	32.3	32.3	32.6
13, C	45.3	45.5	45.9	45.4
14, C	46.7	46.0	45.1	46.7
15, CH_2_	47.7	48.9	45.6	47.8
16, CH	72.7	72.0	74.6	72.9
17, CH	57.0	55.9	56.4	56.9
18, CH_3_	19.0	17.4	17.1	19.0
19, CH_2_	29.8	28.1	27.1	29.8
20, CH	31.5	30.5 (30.8)^[^ ^f^ ^]^	28.9	26.6
21, CH_3_	18.4	17.5 (18.2)^[^ ^f^ ^]^	17.8	17.7
22, CH_2_	33.9	33.9 (33.9)^[^ ^f^ ^]^	35.6	31.2
23, CH_2_	28.6	27.5 (28.8)^[^ ^f^ ^]^	22.4	26.2
24, CH	80.4	79.4 (80.5)^[^ ^f^ ^]^	82.5	75.2
25, C	73.0	72.5	73.0	73.0
26, CH_3_	26.6	24.3	25.1	26.7
27, CH_3_	23.1	23.5	23.4	22.1
28, CH_3_	22.1	21.2	21.2	23.0
29, CH_3_	20.8	19.8	19.8	20.8
30, CH_3_	20.0	18.5	20.3	19.9

^[a]^ 75 MHz.

^[b]^ 175 MHz.

^[c]^ 100 MHz.

^[d]^ CD_3_OD.

^[e]^ CH.

^[f]^ CDCl_3_.

Compound **8** had the molecular formula C_30_H_50_O_5_ based on its HRDARTMS and ^13^C NMR data. Its IR spectrum exhibited absorption bands for hydroxy (3351 cm^−1^) and carbonyl (1702 cm^−1^) groups. The NMR spectra showed the resonances of a cycloartane derivative, similar to those of **7** (Tables 1 and 2), with an additional proton geminal to a hydroxy group at δ_H_ 3.56 (ddd 10.5, 9.1, 3.5; δ_C_ 69.7). Correlations of this resonance in the COSY experiment, with H_2_‐6 (δ_H_ 1.71 m, 6α and 1.18 m, 6β) and H‐8 (δ_H_ 1.78, d 9.1 Hz), and in the HMBC spectrum, with C‐5 (δ_C_ 47.0), C‐8 (δ_C_ 54.2), and C‐14 (δ_C_ 46.0), allowed to locate the hydroxy group at C‐7. Nuclear overhauser effect spectroscopy (NOESY) correlations between H‐7, H‐5 (δ_H_ 1.93, dd 13.3, 4.2 Hz), H‐6α (δ_H_ 1.71 m), CH_3_‐30 (δ_H_ 0.98 s), and H‐15α (δ_H_ 2.26, dd 14.0, 5.6 Hz) defined the β‐orientation of the hydroxy group at C‐7. Compound **8** also likely had a 24*S* configuration, as the resonances of the side chain were very similar to those of compound **7** (Table 2), and its electronic circular dichroism (ECD) spectrum showed the same pattern as compounds **7–9,** with a negative Cotton effect at λ 300 nm.

**FIGURE 2:**
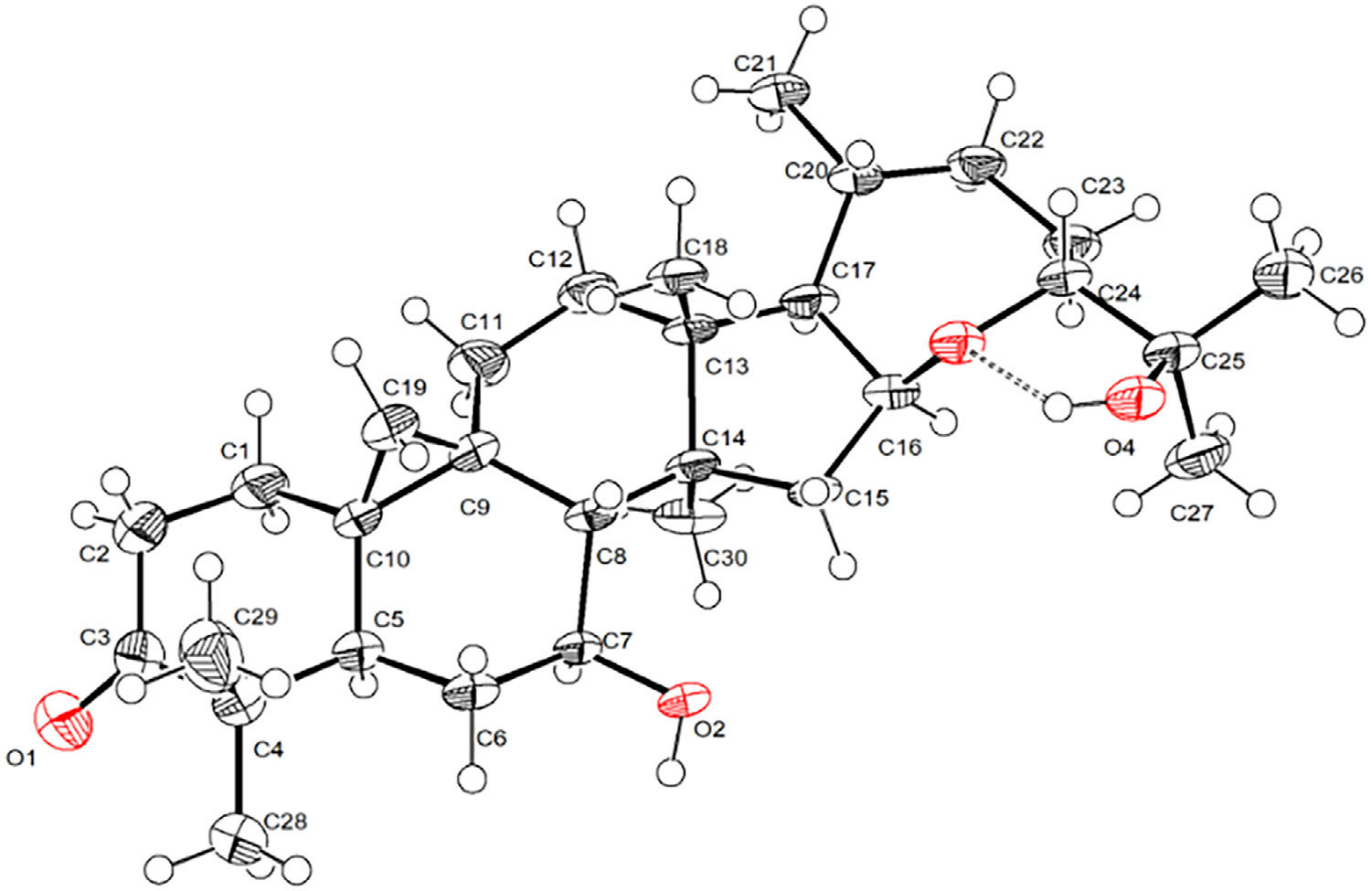
ORTEP drawing of compound **9**.

Compound **9** exhibited the molecular formula C_30_H_48_O_4_, confirmed by HRDARTMS and ^13^C NMR analyses. The IR spectrum showed the hydroxy (3432 cm^−1^) and carbonyl (1701 cm^−1^) groups. The ^1^H NMR spectrum (Table 1), in addition to the characteristic resonances of a cycloartane‐type compound, revealed oxymethine protons of H‐16 (δ_H_ 4.65, q, 7.6 Hz) and H‐24 (δ_H_ 3.53, dd, 12.4, 2.0 Hz), assigned by COSY and HMBC correlations. A third oxygenated methine resonance at δ_H_ 3.62 (ddd 12.6, 9.2, 4.0 Hz) was assigned to H‐7, based on correlations with H_2_‐6 (δ_H_ 1.68 m, 6α and 1.20 m, 6β) and H‐8 (δ_H_ 1.76, d 9.2 Hz) in the COSY spectrum, and with C‐5 (δ_C_ 46.8), C‐8 (δ_C_ 53.6), and C‐14 (δ_C_ 45.1) in the HMBC experiment. An HMBC cross‐peak between H‐16 and C‐24 defined the seven‐membered ring system with an ether bridge between C‐16 and C‐24, as in compounds **1** and **2**. Additionally, a tertiary hydroxyl group at C‐25 (δ_C_ 73.0) was identified in compound **9** from the HMBC spectrum. NOESY correlations of H‐7 with H‐5, H‐6α, H‐15α, and CH_3_‐30 highlighted the β‐orientation of the hydroxy group at C‐7. The relative stereochemistry was confirmed by X‐ray analysis (Figure 2), and the absolute configuration was established based on its ECD spectrum (negative Cotton effect at λ 299 nm), similar to that of argentatin B (**1**), whose absolute configuration has been previously described [31].

### 2.2 In Silico Pesticide‐Likeness Prediction

The pesticide‐likeness properties of compounds **1–16** and betulinic acid (positive control), known for its activity against *S. frugiperda* [16], were calculated (Table 3). The calculated physicochemical parameters included molecular weight (MW < 500 uma), octanol/water coefficient (cLogP ‐1–3), number of hydrogen bond acceptors (HBAs 1–8), number of hydrogen bond donors (HBDs ≤ 2), number of rotatable bonds (RBs ≤ 12), and number of aromatic atoms (AA ≤ 17). These properties were analyzed based on the criteria outlined for identifying potential pesticide candidates [32].

**TABLE 3:** Pesticide‐likeness prediction of compounds **1**–**16**.

Compound	MW	cLogP	HBA	HBD	RB	AA	Tice violations
	150–500	0–5	1‐8	≤2	≤12	≤17	≤1
1	456.708	5.6514	3	1	1	0	1
2	458.724	5.5077	3	2	1	0	1
3	484.718	5.3454	4	0	2	0	1
4	470.691	4.9175	4	1	1	0	0
5	470.691	4.9175	4	1	1	0	0
6	398.585	4.6053	3	0	0	0	0
7	474.723	5.3259	4	3	5	0	2
8	490.722	4.4738	5	4	5	0	1
9	472.707	4.7993	4	2	1	0	0
10	302.456	4.1175	2	1	1	0	0
11	400.557	5.013	4	1	4	0	0
12	300.44	4.013	2	1	1	0	0
13	316.439	3.1609	3	2	1	0	0
14	314.423	3.3046	3	1	1	0	0
15	346.421	1.283	5	2	2	0	0
16	350.409	2.2523	6	1	3	0	0
Betulinic acid (Control)	456.71	6.37	3	2	2	0	1

**MW**: Molecular weight; **clogP**: Octanol/water coefficient; **HBA**: Hydrogen bond acceptors; **HBD**: Hydrogen bond donors; **RB**: Rotatable bonds; **AA**: Aromatic atoms.

According to the Tice rules, a pesticide candidate should have an MW of less than 500 amu. In this study, the MW of the natural products isolated from *V*. *dentata* ranged from 484.72 to 300.44 amu. Compounds **1** and **2** had an MW similar to that of betulinic acid (control), while compounds **6**, **10**, and **12–16** had MWs lower than the control.

cLogP, which reflects the permeability of substances through cell membranes, was also assessed. Compounds **4–6**, **8–10**, and **12–16** had cLogP values of <5. Notably, compound **15** exhibited a lower cLogP (1.28) than the control (6.37). For insecticides, the mean value of cLogP typically ranges from 0.5 to 3.0 [32]. All the substances followed the HBA requirements proposed by Tice's rules, while compounds **1–6** and **9–16** fitted within the HBD interval.

In terms of the number of RB, Compounds **1**, **2**, **4–6**, **9**, **10**, and **12–14** exhibited lower RB values than betulinic acid. A lower RB value indicates poor molecular flexibility, which is favorable for promoting major interactions with the target. Based on these analyses, 15 of the 16 (**1–6** and **8–16**) compounds were considered possible pesticide candidates, as they exhibited one or no violations of Tice's rules.

### 2.3 Evaluation of the Activity of the Isolated Compounds on the Inhibition of AChE

The inhibitory effects of the isolated compounds on AChE were also evaluated. Inhibiting this protein has been an effective strategy for insecticide development because it regulates the acetylcholine level, and specific residues in the insect AChE active sites are promising targets for the development of new insecticides [17]. Compounds showing a strong affinity for AChE can cause rapid paralysis and death in insects, making them candidates for pest control. The results showed weak activity for compounds **1** and **6**, with IC_50_ values of 78.85 ± 6.95 and 53.54 ± 2.53 µM, respectively (Table 4). Previous studies on *ent*‐kaurenoic acid (**10**) reported IC_50_ values from 34.82 ± 0.45 µM [19] (using mouse brain AChE). In our conditions (using *E. electricus* AChE), compound **10** inhibited AChE by only 18.86% at 100 µg/mL during the primary screening (no IC_50_ was calculated). These discrepancies should be due to the different origins of AChE.

**TABLE 4:** Inhibitory activity on acetylcholinesterase (AChE) of compounds **1**–**16**, molecular docking analysis, and activity on *Spodoptera frugiperda*.

	Activity on AchE		Molecular docking analysis		Activity on *S. frugiperda*		
Extract (compound/ligand)	100 µM (%)	IC_50_ µM^[^ ^d^ ^]^	Binding energy (kcal/mol)	Dissociation constant [pM]	Larval weight in mg, (inhibition %)^[^ ^e^ ^]^	Mortality (%)	LC_50_ ppm^[^ ^f^ ^]^
HE	12.19^[^ ^a^ ^]^	nt	nc	nc	72.4 ± 10 (70.74)	14 ± 2.5	7397.85
AE	13.80^[^ ^a^ ^]^	nt	nc	nc	127.8 ± 12.9 (48.36)	37 ± 9.8	2407.64
ME	6.75	nt	nc	nc	106.4 ± 13.4 (56.99)	32 ± 6.3	5362.7
1	67.18	78.85 ± 6.95	−8.70	420 450.0	57.7 ± 7.3 (76.68)	59 ± 10.3	51.61
2	15.60	nt	−8.76	376 180.0	60.5 ± 7.2 (75.55)	40 ± 9.1	84.56
3	20.95	nt	−8.20	972 070.0	nt	nt	nt
4^[^ ^b^ ^]^	10.87	nt	−8.53	765 450.0	nt	nt	nt
5^[^ ^b^ ^]^			−7.96	1 470 000.0	nt	nt	nt
6	78.85	53.54 ± 2.53	−8.89	306 530.0	84.1 ± 10.1 (66.02)	17 ± 3.5	1433.4
7	36.78	nt	−9.40	128 370.0	nt	nt	nt
8	4.25	nt	−9.12	206 760.0	nt	nt	nt
9	−7.03	nt	−8.63	475 140.0	93.3 ± 10.3 (62.30)	42 ± 8.7	99.66
10	18.86	nt	−10.59	17 330.0	77.8 ± 7.3 (68.56)	60 ± 12.4	24.69
11	26.05	nt	−10.49	20 470.0	nt	nt	nt
12	26.41	nt	−11.01	8550.0	nt	nt	nt
13	15.37	nt	−11.59	3200.0	66.3 ± 6.5 (73.21)	59 ± 17.8	62.04
14	2.35	nt	−10.87	10 850.0	nt	nt	nt
15	9.11	nt	−10.94	9560.0	nt	nt	nt
16	14.16	nt	−11.14	6780.0	nt	nt	nt
Galantamine	52.53^[^ ^c^ ^]^	0.32 ± 0.01	−10.32	27 300.0			
Betulinic acid (control)	71.55 [18]	7.07 ± 1.09 [18]	−11.13 [18]	2 450 000.0	129.22 ± 7.2 (47.78)	47 ± 11.5	94.25
Artificial diet					247.5 ± 22.4	7 ± 0.7	

HE: hexane extract, AE: acetone extract, nt: not tested, nc: not calculated,

^[a]^ 100 mg/L,

^[b]^ AChE activity tested as a mixture of **4** and **5**,

^[c]^ galantamine reference compound 0.25 µM,

^[d]^ Half‐inhibitory concentration (IC_50_),

^[e]^ percentage of inhibition of larval weight in relation to the artificial diet control, and

^[f]^ Lethal concentration that killed 50% of the larvae (LC_50_).

### 2.4 Interactions between the Isolated Compounds and AChE Were Assessed Using Molecular Docking Techniques

Molecular docking calculations were performed to evaluate the interaction of ligands **1–16** with AChE. Residues involved in hydrogen bonding and hydrophobic interactions were identified, and the binding energy and dissociation constant were determined (Table 4).

*ent*‐Kaurenes **10** and **13** were found to be the most potent AChE inhibitors, with binding energies of −10.59 kcal/mol and −11.59 kcal/mol, respectively, and these relatively high energies correlated with the observed high mortality of *S. frugiperda* (50% lethal concentration for larval mortality [LC_50_] for **10**: 24.69 ppm and LC_50_ for **13**: 62.04 ppm; positive control: betulinic acid LC_50_ 94.25 ppm) (See Table 4 and discussion below). Docking experiments for the *ent*‐kaurenoid acids **11**, **12**, **14**, and **15** exhibited similar binding energies and dissociation constants, indicating strong affinity and specificity for the enzyme, but unfortunately, these compounds were not available for in vivo experiments.

The cycloartanes **1**, **2**, and **9** also exhibited relatively high binding energies of −8.70, −8.76, and −8.63 kcal/mol, respectively, in the docking experiments, and these values also correlated with the observed in vivo activity (LC_50_ for **1**: 51.61 ppm, LC_50_ for **2**: 84.56 ppm, and LC_50_ for **9**: 99.66 ppm).

**FIGURE 3:**
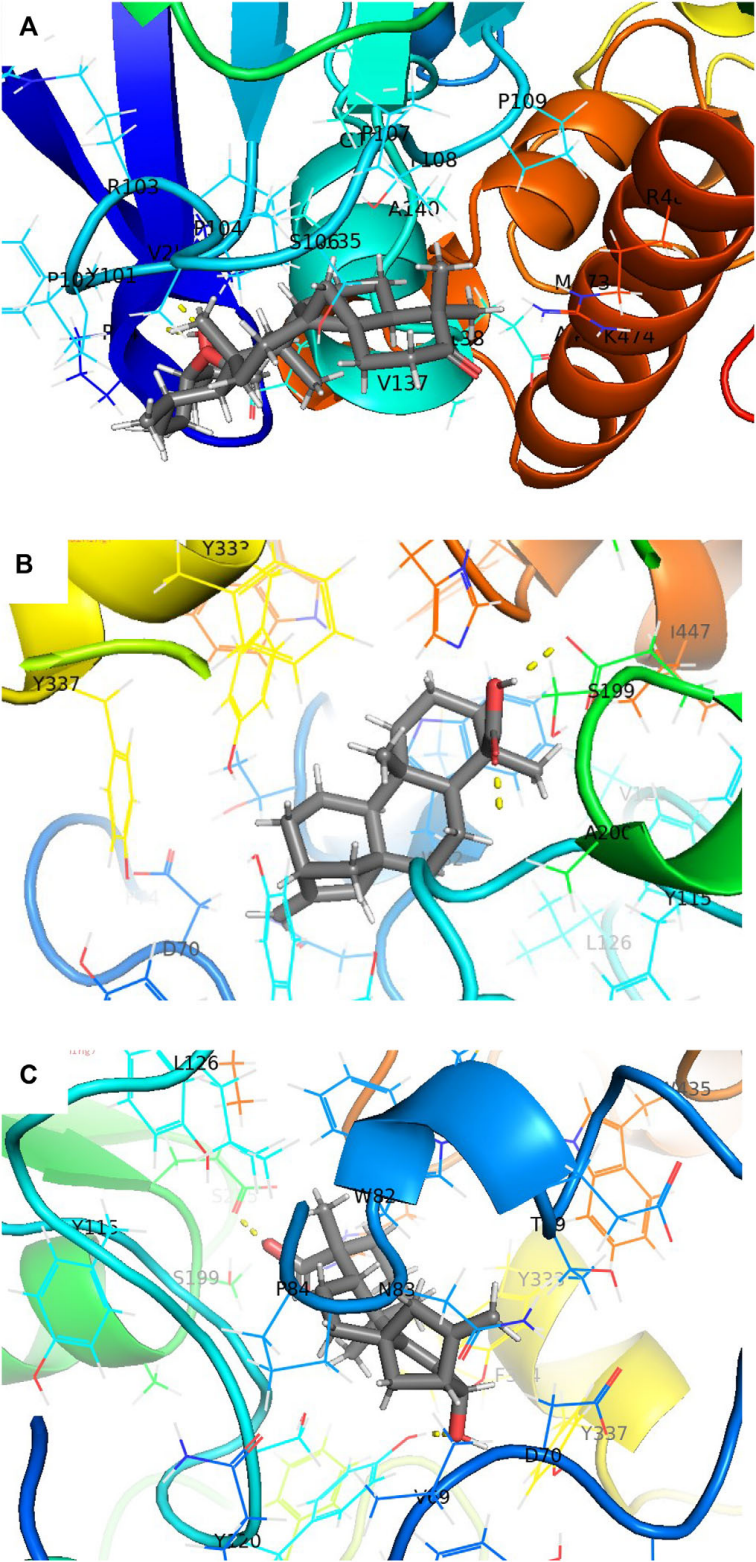
Molecular docking interactions of some compounds tested against acetylcholinesterase (AChE). (A) Argentatin B (**1**), (B) *ent*‐kaurenoic acid (**10**), and (C) 12α‐hydroxy ent‐kaur‐9(11),16‐dien‐19‐oic acid (**13**).

Figure 3 shows several significant molecular interactions for Argentatin B (**1**), *ent*‐kaurenoic acid (**10**) and 12α‐hydroxy *ent*‐kaur‐9(11),16‐dien‐19‐oic acid (**13**). Compound **1** interacts with AChE through hydrogen bonding interactions with P24 and Y101, these residues are not involved in the catalytic site, therefore an allosteric inhibition can explain the activity against the enzyme (Figure 3A). On the other hand, substances **10** and **13** interact with Y337 through hydrophobic contacts (Figure 3B,C; see Supporting Information). Y337 is responsible for maintaining the electrostatic balance of AChE's catalytic cavity, and this interaction is comparable to that observed with betulinic acid (the positive reference used in this work), which has been associated with AChE inhibition [16].

Several amino acids participate in the contacts with the cycloartanes and the *ent*‐kaurenoic acids, including L285, W282, Q287, and E288 among others (see Supporting Information). The interactions shown in Figures 3A–C highlight the extensive range of molecular contacts that significantly influence the binding affinity of the compounds to AChE.

### 2.5 Insectistatic and Insecticide Activities of Extracts, and Compounds **1**, **2**, **6**, **9**, **10**, and **13** on *Spodoptera frugiperda*

#### 2.5.1 Insectistatic Effect

The insecticidal activity of the extracts and compounds **1**, **2**, **6**, **9**, **10**, and **13** (selected based on their availability of 10 mg each for the assay) was tested against *S. frugiperda*. All extracts and compounds inhibited larval weight. Hexane, methanol, and acetone extracts reduced larval weight by 70.74%, 56.99%, and 48.36%, respectively, compared with the negative control (247.52 mg larval weight, Table 4). Compounds **1**, **2**, **6**, **9**, **10**, and **13** clearly showed antifeedant properties showing higher percentages of inhibition of the larval weight in comparison with the reference betulinic acid (47.78% of inhibition) (Table 4).

#### 2.5.2 Insecticide Effect

The results indicated that application of the acetone extract caused 37% mortality, followed by methanol and hexane extracts with 32% and 14% mortality, respectively. Among the pure compounds, *ent*‐kaurenoic acid (**10**) was the most active, followed by argentatin B (**1**), 12α‐hydroxy *ent*‐kaur‐9 (11),16‐dien‐19‐oic acid (**13**), argentatin D (**2**), and 7β‐hydroxy‐argentatin B (**9**). Their LC_50_ values were 24.69, 51.61, 62.04, 84.56, and 99.6 ppm, respectively. Betulinic acid (reference compound) had an LC_50_ of 94.25 ppm (Table 4). These activities are similar to those reported previously [13, 33, 34, 35].

In the in vitro AChE assays, cycloartane **6** exhibited weak activity (IC_50_ of 53.54 µM), followed by cycloartane **1** (IC_50_ 78.85 µM). The same trend was observed in the docking analysis, with compound **6** showing a slightly lower binding energy (– 8.89 kcal/mol) than compound **1** (–8.70 kcal/mol). However, *ent*‐kaurane derivatives (**10–15**) and sesquiterpene lactone **16** were the most competitive inhibitors of AChE, with binding energies between –11.59 and –10.59 kcal/mol. These values were lower than that of galantamine, the reference compound, with a calculated binding energy of –10.32 kcal/mol (Table 4).

Compounds **1**, **2**, **9**, **10**, and **13** displayed LC_50_ mortalities values lower than the LC_50_ of the positive reference (betulinic acid 94.25 ppm) and can be considered bioactive compounds. The molecular docking results on AChE of compounds **10** and **13** correlated with those obtained from the evaluation against *S. furgiperda*: compound **10**, whose affinity energy is among the highest (‐10.59 kcal/mol) presented the highest mortality of the substances evaluated (LC_50_ 24.69 ppm), and compound **13** exhibited a binding energy of ‐11.59 kcal/mol and LC_50_ of 62.04 ppm. Compounds **1** (‐8.70 kcal/mol), **2** (‐8.76 kcal/mol), and **9** (‐8.63 kcal/mol) displayed LC_50_ of 51.61, 84.56, and 99.6 ppm, respectively. Thus, considering the mortality LC values, the relative activity of the isolated compounds was determined to be **10**> **1**> **13**> **2**> **9**.

## 3 Conclusions

The chemical study of *V. dentata*, a species appreciated as a melliferous plant, led to the isolation of 20 compounds, including cycloartanes and *ent*‐kauranes as the main and characteristic secondary metabolites, with three new compounds identified (**7–9**). The majority of the isolated compounds were evaluated through in silico studies (pesticide‐likeness properties, indicating the absence of Tice's rules violations; molecular docking, recognizing the interactions of bioactive compounds with Y337 in the catalytic site of AChE); in vitro assays (inhibition of the enzyme AChE by some compounds), and in vivo studies (insectistatic and insecticide activities against *S. frugiperda*). Among the compounds evaluated, argentatin B (**1**) displayed the greatest inhibitory effect on *S. frugiperda* larval weight. This compound, along with cycloartanes **2** and **9**, and the *ent*‐kaurenoic acids **10** and **13** had clear effects on insect mortality. The results of the docking calculations with the AChE enzyme correlated with the mortality results against the fall armyworm, and demonstrated that argentatins (**1**, **2**, and **9**) and *ent*‐kaurenoic acids (**10** and **13**) have the potential to control *S. frugiperda*.

## 4 Experimental

### 4.1 General Experimental Procedures

Melting points were determined on a Fisher‐Johns apparatus and were uncorrected. Optical rotations were obtained on a Perkin‐Elmer 343 polarimeter. IR spectra were recorded on a Thermo Scientific Nicolet iS50 FT‐IR spectrometer. ECD was obtained on a Jasco J‐720 CD spectropolarimeter. 1D and 2D NMR spectra were obtained on a Bruker Avance (F) 300 MHZ, a Bruker Avance III 400 MHz, or a Bruker AVANCE III HD 700 MHz spectrometer with tetramethylsilane (TMS) as the internal standard. X‐ray diffraction analysis was performed on an Xcalibur Atlas Gemini diffractometer with a Mo X‐ray source. The DART‐MS was performed using a JEOL AccuTOF JMS‐T100LC DART. Vacuum column chromatography (VCC) was performed under vacuum on silica gel G 60 (Merck, Darmstadt, Germany). Flash column chromatography (FCC) was performed on silica gel 230‐400 mesh (Macherey‐Nagel, Germany). Analytical TLC was performed on Si gel 60 GF_254_ or RP‐18 W/UV_254_ (10–40) µm, Macherey‐Nagel, Germany) and preparative TLC on Si gel GF_254_ layer thickness 2.0 mm or RP‐18 W/UV_254_ layer thickness 1.0 mm, using 10 × 20 cm plates.

### 4.2 Plant Material

Aerial parts of *V. dentata* (Cav.) Spreng. were collected along the road Tequisquiapan‐Queretaro, 30 km after Tequisquiapan (Hwy 200), Queretaro State, México, in October 2018 and authenticated by Prof. José Luis Villaseñor. A voucher specimen (MEXU 1473183) was deposited at the National Herbarium (Instituto de Biología, UNAM, México).

### 4.3 *Spodoptera frugiperda* (J.E. Smith)

The larvae of *S. frugiperda* were reared under laboratory conditions at the Departamento de Desarrollo de Productos Bióticos del Instituto Politécnico Nacional (Yautepec, Morelos). The diet formula was 800 mL distilled water, 60 g diet (Product No. F0635; S.W. Corn Borer, Bio‐Serv, Frenchtown, NJ, USA), 20 g sterile corn cob, 100 g ground corn, 40 g brewer's yeast, 10 g vitamins (Lepidoptera fortification blend, Bio‐Serv, Flemington, NJ), 10 g agar, 1.7 g sorbic acid, 1.7 g methyl p‐hydroxybenzoate, and 0.6 g neomycin sulfate. Insects were maintained in a climate chamber set at 25 ± 2°C, 60 ± 5% RH, and 12:12 h L:D [36].

### 4.4 Extraction and Isolation

The dried and ground aerial parts of *V. dentata* (800 g) were successively extracted with hexane, acetone, and MeOH at room temperature to obtain the respective extracts. The hexane extract (44 g) was fractionated on silica gel G 60 using VCC and eluted with a hexane‐EtOAc gradient system to obtain argentatin B (**1**, colorless prisms [hexane‐acetone] melting point [mp] 175–176°C, 1.2 g) and argentatin D (**2**, colorless prisms [hexane acetone] mp 234–235°C, 70 mg), from fractions eluted with hexane‐EtOAc 17:3 and 4:1, respectively, and mixtures A‐I. Mixture A (8.0 g), obtained with hexane‐EtOAc (19:1), after purification via a silica gel 230–400 mesh FCC eluted with hexane‐EtOAc 19‐1 afforded 1.8 g of a mixture of *ent*‐kaurenoic and grandiflorenic acids (4:1), which by recrystallization in methanol gave *ent‐*kaurenoic acid (**10**, colorless prisms [MeOH] mp 176–177°C, [α]^25^_D_ = _–_102, *c* = 0.13, EtOH, 560 mg), 60 mg of its mother liquors were separated by preparative RP TLC (MeOH‐H_2_O 4:1 × 2) to obtain 37 mg of **10** and 10 mg of grandiflorenic acid (**12**, colorless prisms [MeOH] mp 156–158°C, [α]^25^_D_ = + 33, *c* = 0.10, EtOH). Mixture B (2.76 g) obtained using hexane‐EtOAc 19‐1 gave mixtures B1 and B2. Mixture B1 (150 mg) was submitted to two successive FCCs (eluted with hexane‐EtOAc‐19‐1 and hexane‐acetone 19‐1, respectively) to afford 10 mg of 15‐angeloylgrandifloric (**11**, colorless prisms (hexane ‐acetone) mp 198–200°C, [α]^25^_D_ = _–_68, *c* = 0.13, CHCl_3_). Mixture B2 (372 mg) was purified by FCC (benzene‐acetone 97:3) to obtain verbenol (colorless oil, 27 mg). Mixture C (1.9 g), obtained with hexane‐EtOAc (9:1), was purified by FCC (hexane‐EtOAc 9:1) to yield compound **1** (92 mg) and the 1:1 mixture of β‐sitosterol and stigmasterol (45 mg). Mixture D (2.5 g), obtained with hexane‐EtOAc (17:3), was purified through an FCC (hexane‐EtOAc 9:1) to obtain **1** (320 mg). Mixture E (1.08 g) obtained with hexane‐EtOAc (17:3) was subjected to FCC (hexane‐acetone 9:1) to afford compounds **4** and **5** as a mixture (120 mg) and fraction E1. Fraction E1 (317 mg) by FCC (hexane‐acetone 17:3) gave **4** and **5** mixtures (22 mg), and **2** (10 mg). Mixture F (1.82 g) obtained with hexane‐EtOAc (4:1) was purified by FCC (hexane‐acetone 9:1) to yield **2** (80 mg). Mixture G (630 mg) obtained with hexane‐EtOAc 7:3 was purified by FCC (hexane‐acetone 17:3) to yield compound **2** (64 mg) and mixture G1. Mixture G1 (525 mg) after two successive FCC (CH_2_Cl_2_‐acetone 95:5 and hexane acetone 4:1, respectively) gave compound **14** (15 mg, colorless prisms (MeOH) mp: 128–130°C, ${\left[a\right]}_{D}^{25}$ = + 35, *c* = 0.10, CHCl_3_, 15 mg). Mixture H (1.6 g) eluted with hexane‐EtOAc 7:3 gave compound **13** (colorless needles (MeOH) mp: 187‐190°C, ${\left[a\right]}_{D}^{25}$ = + 83, *c* = 0.13, CHCl_3_, 262 mg) and mixture H1. Mixture H1 (395 mg) was purified by FCC (CH_2_Cl_2_‐acetone 19:1) to obtain a solid, which, by crystallization with isopropyl ether, afforded diversifolin (**16**, amorphous powder, 28 mg). Mixture I (964 mg) was eluted with hexane‐EtOAc 3:2 and purified by FCC (CH_2_Cl_2_‐acetone 9:1) to produce mixtures I1 and I2. Mixture I1 (189 mg) by FCC (CH_2_Cl_2_‐acetone 9:1) followed by preparative TLC (CH_2_Cl_2_‐acetone 17:3) afforded compound **9** (25 mg). Mixture I2 (195 mg) was purified by preparative TLC (hexane‐acetone 7:3) afforded compound **7** (13 mg). The acetone extract (43 g) was fractionated in a VCC (hexane‐EtOAc gradient system) to obtain mixtures J‐P. Mixture J (275 mg) obtained with hexane‐EtOAc 19:1 was purified with FCC (hexane‐acetone 19:1) to obtain caryophyllene oxide (colorless oil, 27 mg). Mixture K (920 mg), obtained with hexane‐EtOAc 9:1, was treated with charcoal/acetone followed by FCC (hexane‐EtOAc 4:1) to give compounds **1** (103 mg), **2** (31 mg), and **3** (18 mg). Mixture L (1.1 g), eluted with hexane‐EtOAc (17:3), was treated with activated charcoal/acetone to obtain an amber oil, which was submitted to a VCC eluted with a gradient of hexane‐EtOAc 19‐1 to 9:1 to obtain compounds **1** (15 mg), **2** (40 mg), and **6** (32 mg). Mixture M (209 mg) was treated with charcoal/acetone followed by FCC (hexane‐EtOAc 4:1) to obtain **6** (15 mg). Mixture N (4.3 g) obtained with hexane‐EtOAc (17:3) after charcoal/acetone treatment was purified by VCC (hexane‐acetone gradient system) to yield compound **13** (115 mg) and mixtures N1‐N2. Mixture N1 (610 mg) was subjected to FCC (hexane‐EtOAc 7:3) to obtain compounds **13** (56.7 mg) and **6** (5.5 mg). Mixture N2 (355 mg) was purified by FCC (hexane‐acetone 7:3) to yield compounds **13** (10 mg) and **15** (amorphous powder, [α]^25^_D_ =_–_16.4, *c* = 0.11, CHCl_3_, 16 mg). Mixture O (2.45 g), obtained with hexane‐EtOAc 4:1, after two successive FCCs (hexane‐EtOAc 3:2 and CH_2_Cl_2_‐acetone 4:1, respectively), yielded compound **9** (26 mg). Mixture P (3.8 g), obtained with hexane‐EtOAc 1:1, was purified by VCC (hexane‐EtOAc), followed by two successive FCC (EtOAc‐MeOH 9:1 and CH_2_Cl_2_‐MeOH 9:1, respectively) to obtain compound **8** (11 mg). From the methanol extract (60 g), a mixture of *ent*‐kaurenoic (**10**) and grandiflorenic acids (**12**, 1.5 g) was characterized, and β‐sitosteryl β‐D‐glucopyranoside (50 mg) and sucrose (80 mg) were isolated.

### 4.5 Spectral Data for the New Compounds

Argentatin B (**1**): CD (CHCl_3_) Δ*ε*_max_: + 0.233_209,_ ‐ 0.321_232_, ‐ 3.242_297_ (*c* 2.2×10^−3^ M).

24‐*Epi‐*argentatin C (**7**): colorless needles (hexane‐acetone) mp 145–146°C, [α]^25^_D_ ‐8.2 (*c* 0.11, CHCl_3_); IR (ATR) *ν*_max_ 3402, 1701 cm^−1^; CD (CHCl_3_) Δ*ε*_max_: +0.321_213_,–0.111_233_, –1.963_299_ (*c* 2.1×10^−3^ M); ^1^H NMR data, see Table 1; ^13^C NMR data, see Table 2; DART^+^ *m/z* 475 [M + H]^+^ (15), 457 (50), 439 (100), 421 (30); HRDARTMS *m/z* 475.37938 [M + H]^+^ (C_30_H_51_O_4_ requires 475.37873).

7β‐Hydroxy‐24‐*epi*‐argentatin C (**8**): white amorphous powder, [α]^25^_D_ + 5.8 (*c* 0.12, MeOH); IR (ATR) *ν*_max_ 3352, 1702 cm^−1^; CD (CHCl_3_) Δ*ε_l_*_max_: ‐0.191_217_, ‐0.260_237_, ‐1432_300_ (*c* 2.4×10^−3^ M); ^1^H NMR data, see Table 1; ^13^C NMR data, see Table 2; DART^+^ *m/z* 491 [M + H]^+^ (10), 473 (25), 455 (90), 437 (100), 419 (30); HRDARTMS *m/z* 491.37583 [M + H]^+^ (C_30_H_51_O_5_ requires 491.37365).

7β‐Hydroxyargentatin B (**9**): colorless prisms (hexane‐acetone), mp 175–176°C, [α]^25^_D–_62.5 (*c* 0.16, CHCl_3_); IR (ATR) *ν*_max_ 3433, 1701 cm^−1^; CD (CHCl_3_) Δ*ε*_max_: + 0.072_209_, –0.420_233_,–3.725_299_, (*c* 3.4×10^−3^ M); ^1^H NMR data, see Table 1; ^13^C NMR data, see Table 2; DART^+^ *m/z* 473 [M + H]^+^ (10), 455 (50), 437 (100), 419 (30); HRDARTMS *m/z* 473.36445 [M + H]^+^ (C_30_H_49_O_4_ requires 473.36308).

Crystal data for compound **9**: C_30_H_48_O_4_, Mr 472.68, monoclinic, space group P21, a = 15.673(4) Å, α = 90o, b = 6.0278(11) Å, β = 117.56(3)o, c = 15.800(4) Å; γ = 90o, V = 1323.2(6) Å3, Z = 2, Dc = 1.186 Mg/m3, F(000) = 520; crystal dimensions/shape/color 0.3560 × 0.1942 × 0.1406 mm3/prism/colorless. Reflections collected 9441, independent reflections 5333 [R(int) = 0.0718]; final R indices [I > 2s(I)] R1 = 0.0762, wR2 = 0.1611; R indices (all data) R = 0.1145, wR2 = 0.1907. Absolute structure parameter 1.5 (10). The deposition number 2445540 for compound **9** contains the supplementary crystallographic data for this work. These data are provided free of charge by the joint Cambridge Crystallographic Data Center and Fachinformationszentrum Karlsruhe http://www.ccdc.cam.ac.uk/structures Access Structures service.

Argentatin C (**17**): colorless prisms (hexane‐acetone) mp 175–176°C, [α]^25^_D_ + 5.7 (*c* 0.10, CHCl_3_); CD (CHCl_3_) Δ*ε*_max_: +0.219_213_,–0.129_251,_ –1.547_299_ (*c* 2.5×10^−3^ M); ^1^H NMR data, see Table 1; ^13^C NMR data, see Table 2.

### 4.6 In Silico Prediction of Pesticide‐likeness Properties

The simplified molecular‐input line‐entry system format (SMILES) was obtained for compounds **1**–**16** and for betulinic acid (control). The chemical structures of **1**–**16** were drawn using ChemDraw software, subsequently transformed to SMILES format, and saved in a .csv Excel document. Then, this was exported to Data Warrior v.5.2.1 software [37] to calculate the following physicochemical properties: MW, cLogP, HBA, HBD, RBs, and the number of AAs.

### 4.7 In Vitro AChE Assay

The AChE inhibitory activity of the isolated compounds was determined by Ellman's method [38], as previously reported,[39] using AChE isolated from *Electrophorus electricus*. Primary screening of compounds **1–16** was performed using 1, 10, and 100 µM concentrations of each compound; samples with less than 50% inhibition at 100 µM were considered non‐active. Galantamine and betulinic acid were used as positive controls. The reported IC_50_ values are the average of five independent experiments.

### 4.8 Docking Methodology

The ligands were initially optimized using Gaussian16 software [40], employing the density functional theory (DFT) with the hybrid density functional B3LYP [41]. Subsequently, the FASTA file of the AChE, ID:1C20, protein of *Electrophorus electricus* was retrieved from the Protein Data Bank (PDB) [42]. For molecular homology and docking calculations, the YASARA [43] and WHAT IF [44] software packages were used with the AutoDockLG [45] algorithm. The molecular docking was performed specifically in one domain of AChE because the two domains are identical. A total of 50 docking runs were performed to evaluate the reproducibility and reliability of the results. This methodology aimed to identify the optimal interaction between the ligand and AChE via different conformations of the ligand. Analysis of hydrogen bonding and hydrophobic interactions, as well as visualization of ligand‐protein interactions, were conducted using PyMOL software [46].

### 4.9 Biological Activity Against *Spodoptera frugiperda* (J.E. Smith)

The insecticidal properties of methanol, hexane, and acetone extracts and of compounds **1**, **2**, **6**, **9**, **10**, and **13** were evaluated in an artificial diet in neonatal larvae following the procedure described in the literature.[47, 48] The extracts and pure compounds were solubilized in methanol:dimethyl sulfoxide 95:5 and evaluated in a range of 500–2500 and 12.5–100 mg/kg (ppm), respectively. The experiments were carried out in plastic containers with lids measuring 3.0 × 3.5 cm in height and diameter; each larva was the experimental unit and replicated thirty times in triplicate. The dependent variables were the decrease in larval weight gain (mg) and larval mortality (%).

### 4.10 Statistical Analysis

The data were presented as the mean ± standard deviation. The test for normality (Shapiro–Wilk–W) and homoscedasticity (Bartlett test) were performed for all measured variables. One‐way to two‐way analysis of variance was performed to identify potential differences among treatments using Statistix 8.0 (Analytical Software, Florida, USA) [49]. Probit analysis was used to calculate the LC_50_ values using the JMP statistical software package ver. 11 [50].

## Author Contributions

**Amira Arciniegas**: isolation and identification of compounds, and writing of the original draft. **Olivia Pérez‐Valera**: pest‐likeness predictions, data curation, and writing the final version. **Simón Hernández Ortega**: X‐ray determination of structure; **Antonio Nieto Camacho,** acetylcholinesterase assays and data curation. **Israel Valencia**: docking studies and review. **Joel Daniel Castañeda‐Espinoza**: *Spodoptera frugiperda* assays. **Rodolfo Figueroa Brito**: writing, review, infrastructure and resources for *S. frugiperda* assays. **José Luis Villaseñor**: collection, identification and registry of the plant and review. **Guillermo Delgado**: research design, data analysis, writing, review, infrastructure and resources.

## Conflicts of Interest

The authors declare no conflicts of interest.

## Supporting information



Supporting Information data associated with this article (^1^H, ^13^C, and 2D NMR spectra of compounds **7–9**, X‐ray data, and docking results) are available on the www under https://doi.org/10.1002/MS‐number.

## Data Availability

Data supporting the findings are available from the corresponding author upon request.
