# Supplementary material for: New Terpenoids from Viguiera dentata: In Silico Pesticide‐Likeness Properties, Acetylcholinesterase Inhibition, Molecular Docking, and Evaluation against Spodoptera frugiperda
Source: Chem Biodivers. 2025 May 6;22(9):e202500917. doi: 10.1002/cbdv.202500917 (PMC12435401; doi:10.1002/cbdv.202500917)
Supplement: Supplementary file 1 — Supporting Information data associated with this article (1H, 13C, and 2D NMR spectra of compounds 7–9, X‐ray data, and docking results) are available on the www under https://doi.org/10.1002/MS‐number. [file CBDV-22-e202500917-s001.pdf]

Supporting information

Content

1.0 Spectra of compounds isolated from *V. dentata* 3

S1. IR spectrum of compound **7**. 3

S2. ^{1}H NMR spectrum of compound **7**. 4

S3. ^{13}C NMR spectrum of compound **7** 5

S4. COSY spectrum of compound **7**. 6

S5. HSQC spectrum of compound **7** 7

S6. HMBC spectrum of compound **7**. 8

S7. Expansion of HMBC spectrum of compound **7**. 9

S8. NOESY spectrum of compound **7**. 10

S9. HRMS spectrum of compound **7**. 11

S10. IR spectrum of compound **8**. 12

S11. ^{1}H NMR spectrum of compound **8**. 13

S12. ^{13}C NMR spectrum of compound **8**. 14

S13. COSY spectrum of compound **8**. 15

S14. HMBC spectrum of compound **8**. 16

S17. HRMS spectrum of compound **8**. 17

S18. IR spectrum of compound **9**. 18

S19. ^{1}H NMR spectrum of compound **9**. 19

S20**.** ^{13}C NMR spectrum of compound **9**. 20

S21. COSY spectrum of compound **9**. 21

S22. HSQC spectrum of compound **9** 22

S23. HMBC spectrum of compound **9**. 23

S24. NOESY spectrum of compound **9**. 24

S25. HRMS spectrum of compound **9**. 25

Crystal Data and Structure Refinement of compound **9**. 26

S26. ^{1}H NMR spectrum of compound **17**. 27

S27. ^{13}C NMR spectrum of compound **17**. 28

S28. COSY spectrum of compound **17**. 29
S29. HSQC spectrum of compound **17**........ 30
S30. HMBC spectrum of compound **17**. 31
S31. HMBC expansion of compound **17**........ 32
S32. NOESY spectrum of compound **17**. 33
2. Molecular docking 34
Figure S1. Molecular docking of compound **1** with acetylcholinesterase. 34
Figure S2. Molecular docking of compound **2** with acetylcholinesterase. 34
Figure S3. Molecular docking of compound **3** with acetylcholinesterase. 34
Figure S4. Molecular docking of compound **4** with acetylcholinesterase. 35
Figure S5. Molecular docking of compound **5** with acetylcholinesterase. 35
Figure S6. Molecular docking of compound **6** with acetylcholinesterase. 35
Figure S7. Molecular docking of compound **7** with acetylcholinesterase. 36
Figure S8. Molecular docking of compound 8 with acetylcholinesterase 36
Figure S9. Molecular docking of compound **9** with acetylcholinesterase. 36
Figure S10. Molecular docking of compound 10 with acetylcholinesterase 37
Figure S11. Molecular docking of compound **12** with acetylcholinesterase. 37
Figure S12. Molecular docking of compound **13** with acetylcholinesterase. 37
Figure S13. Molecular docking of compound **14** with acetylcholinesterase. 38
Figure S14. Molecular docking of compound **15** with acetylcholinesterase. 38
Figure S15. Molecular docking of compound **16** with acetylcholinesterase. 38
Table S1. Molecular Docking Analysis of Ligands Targeting Acetylcholinesterase for Potential Insecticidal Activity 39

1.0 Spectra of compounds isolated from *V. dentata*

S1. IR spectrum of compound **7**.

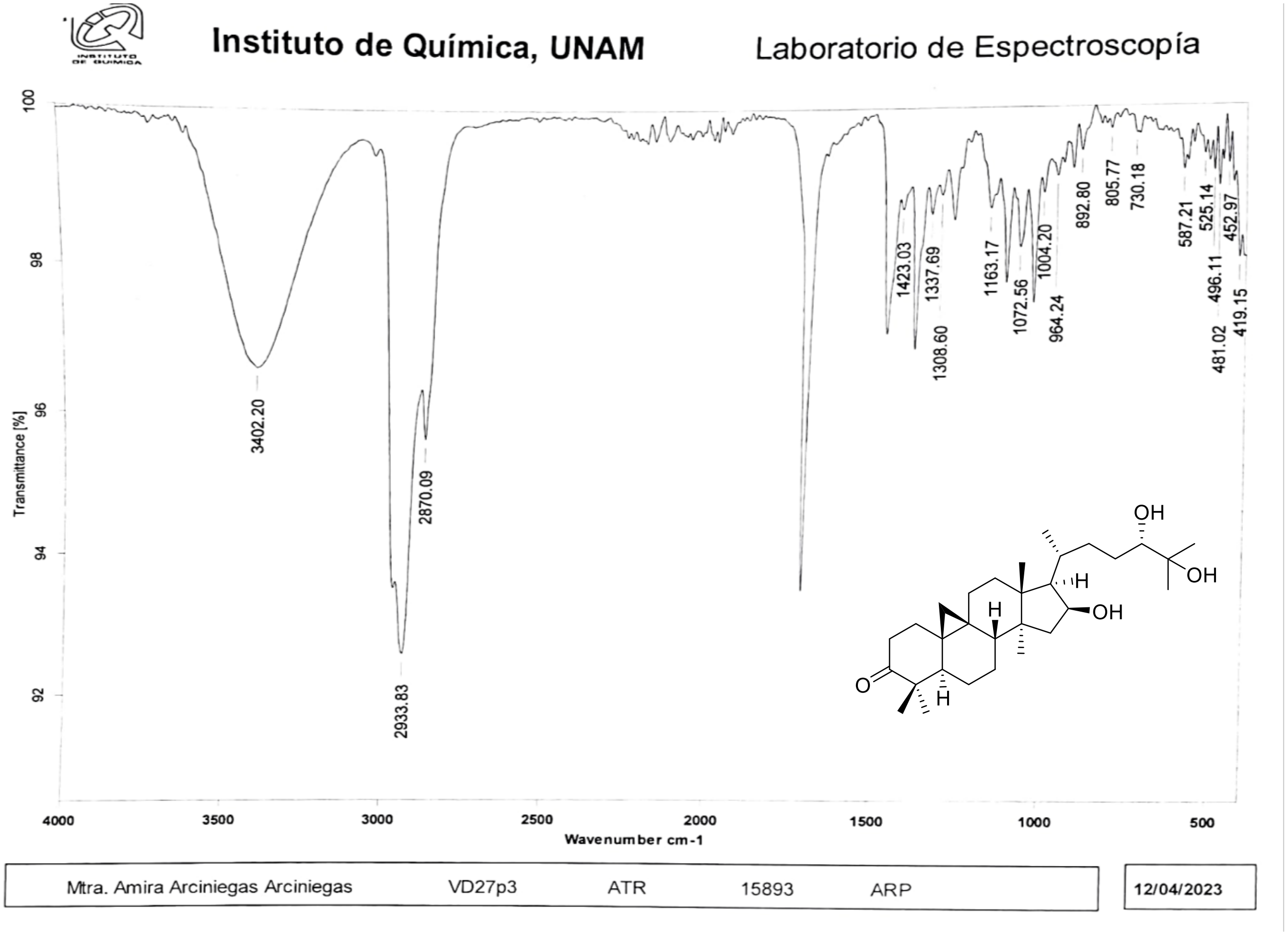

S2. ^{1}H NMR spectrum of compound **7**.

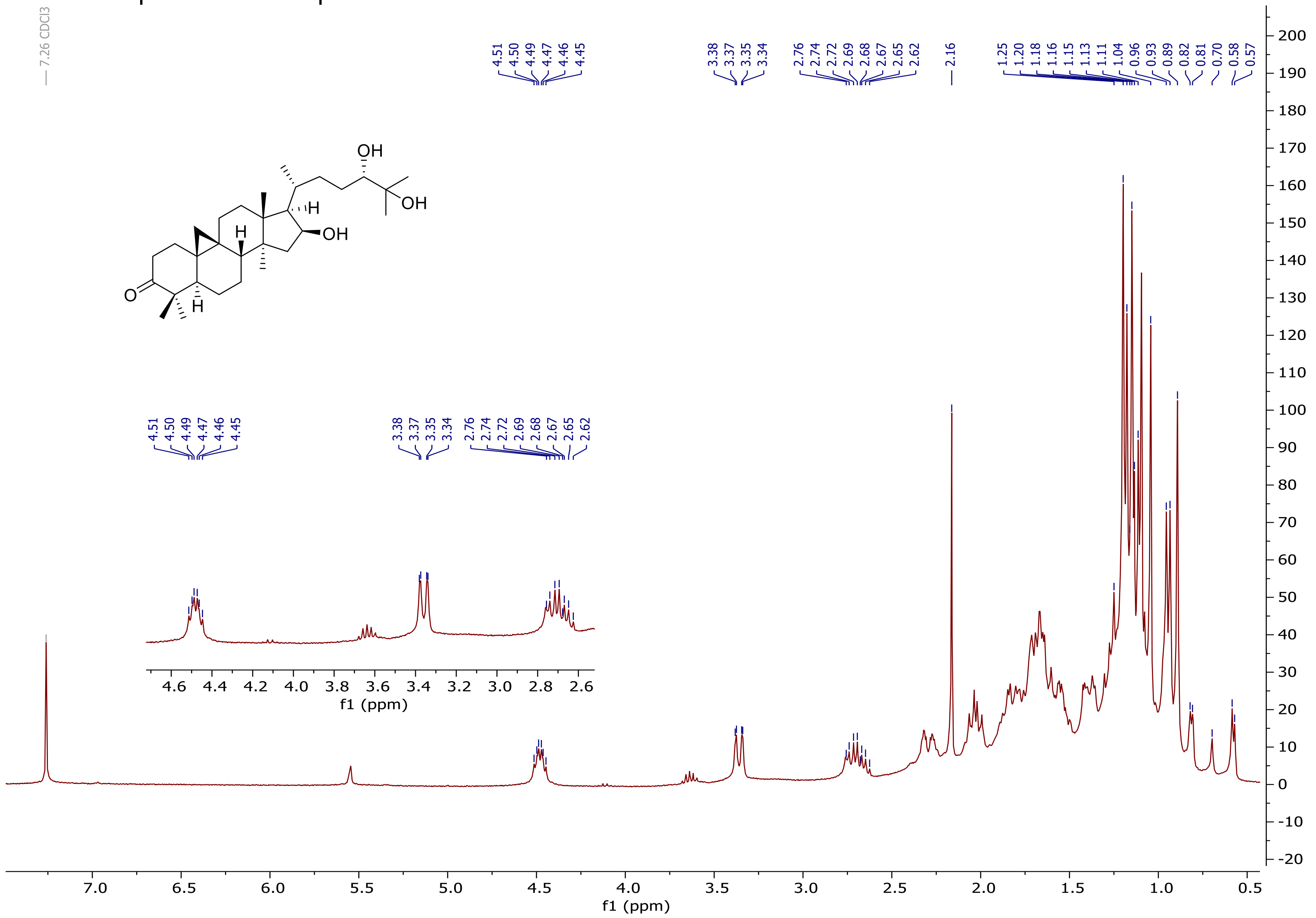

S3. ^{13}C NMR spectrum of compound **7**.

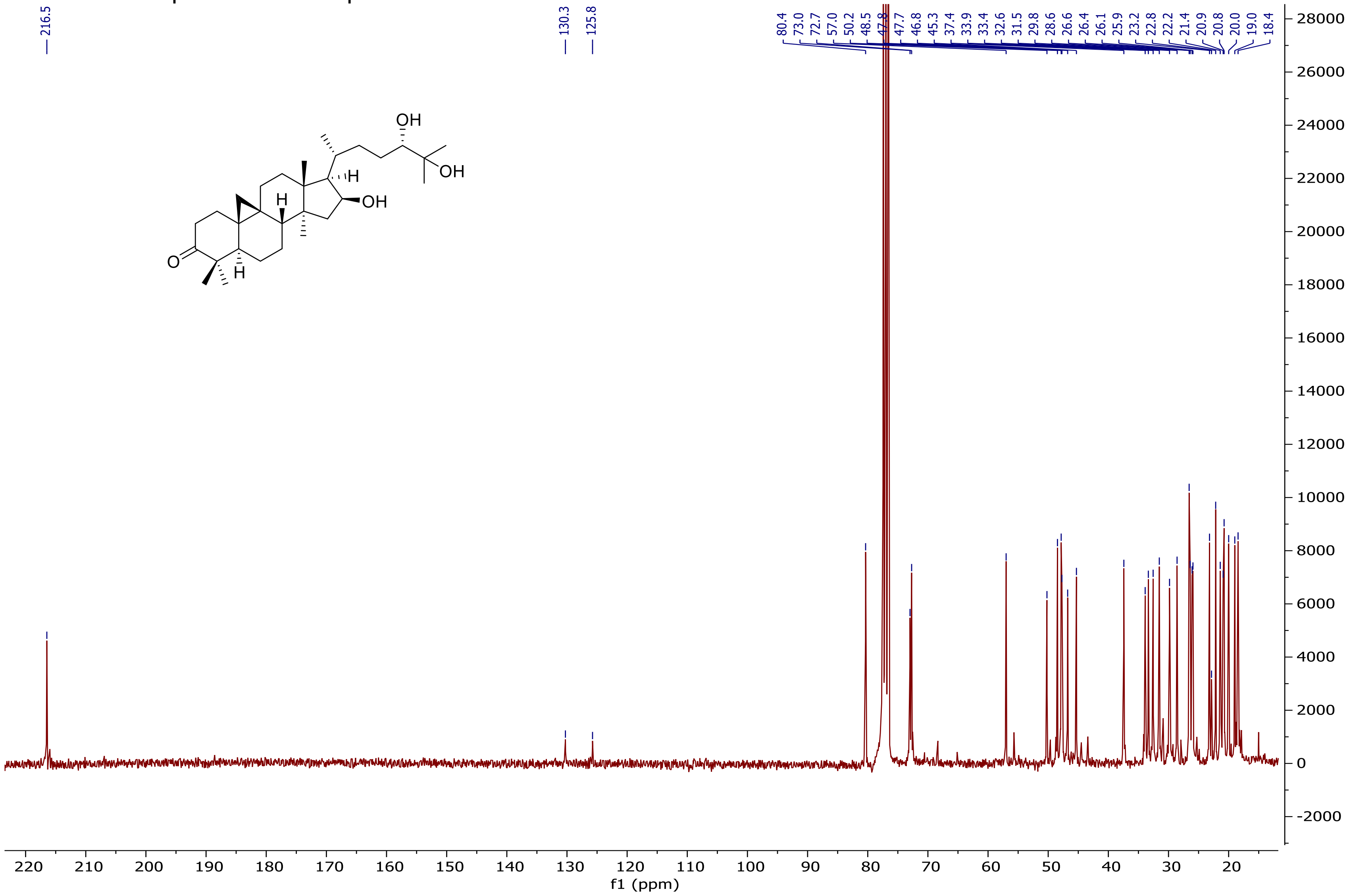

S4. COSY spectrum of compound **7**.

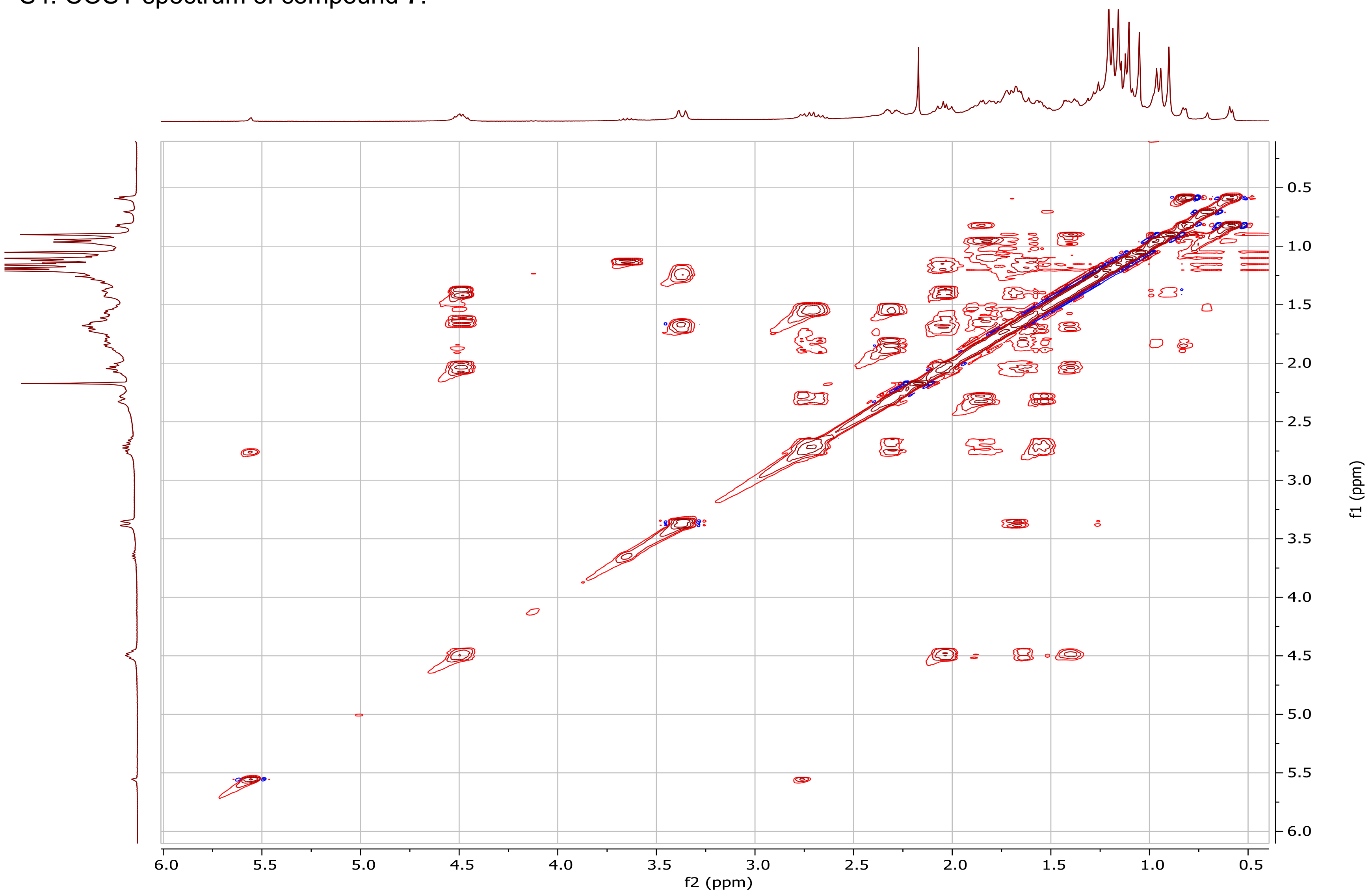

S5. HSQC spectrum of compound **7**.

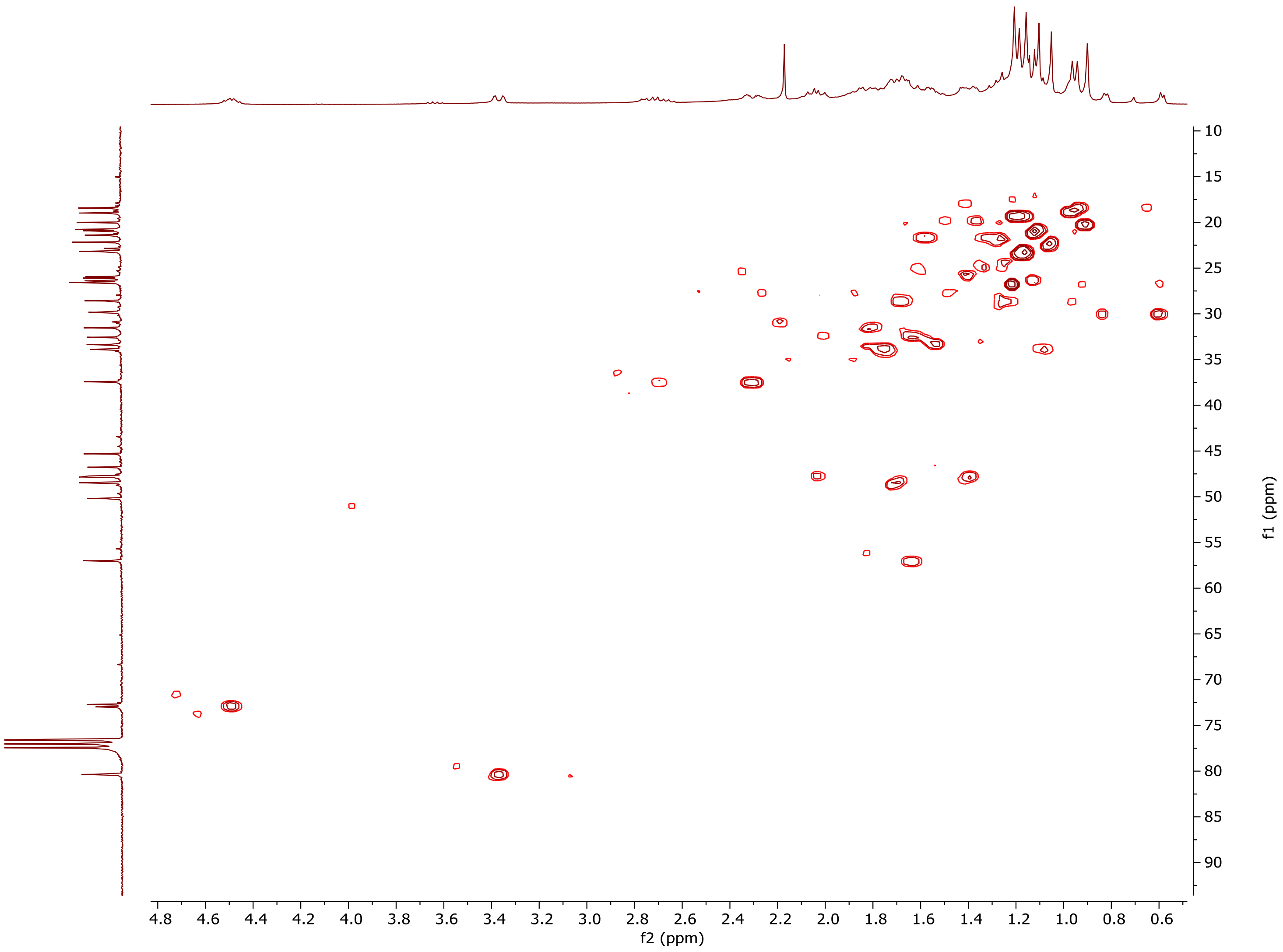

S6. HMBC spectrum of compound **7**.

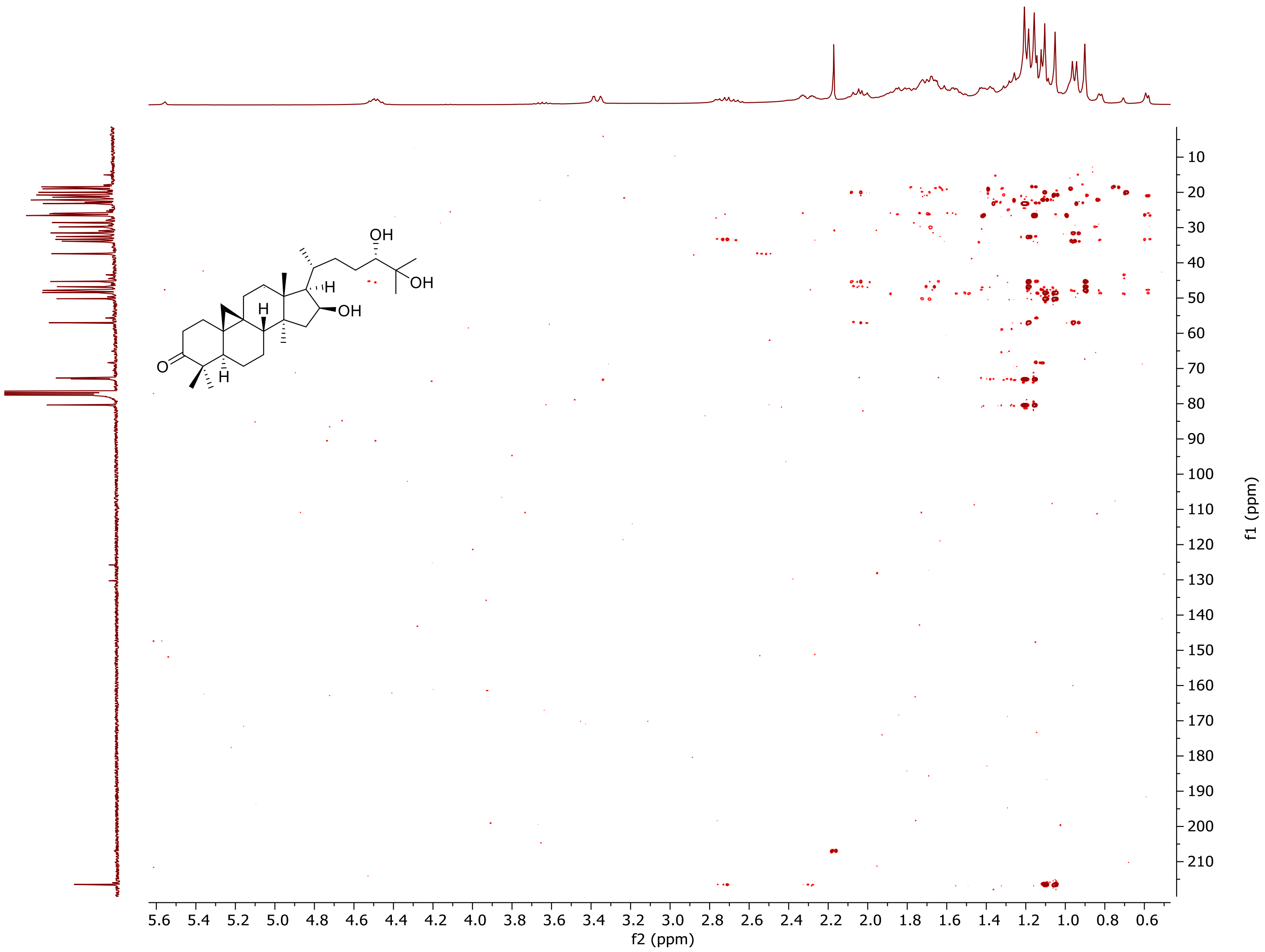

S7. Expansion of HMBC spectrum of compound **7**.

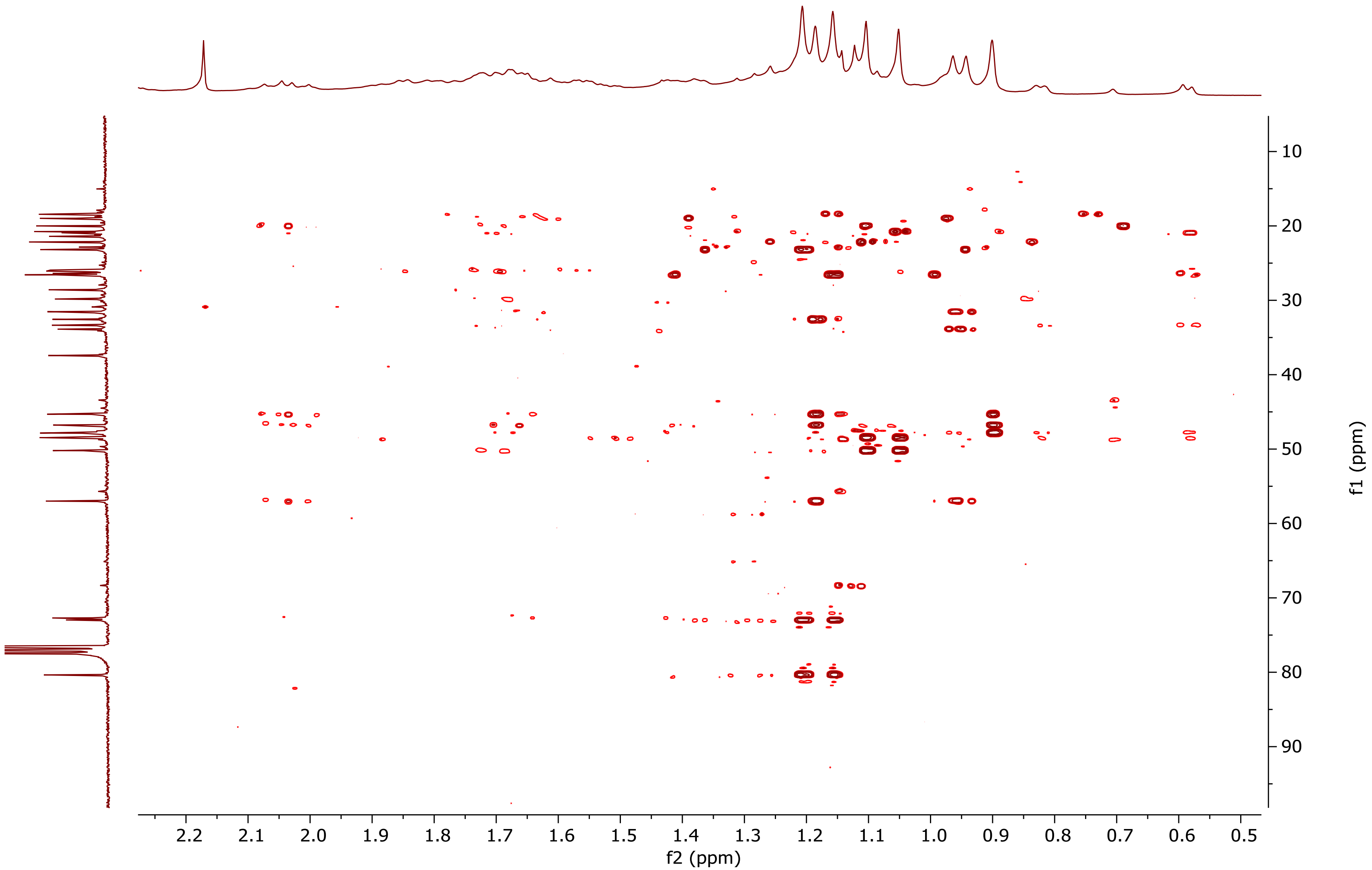

S8. NOESY spectrum of compound **7**.

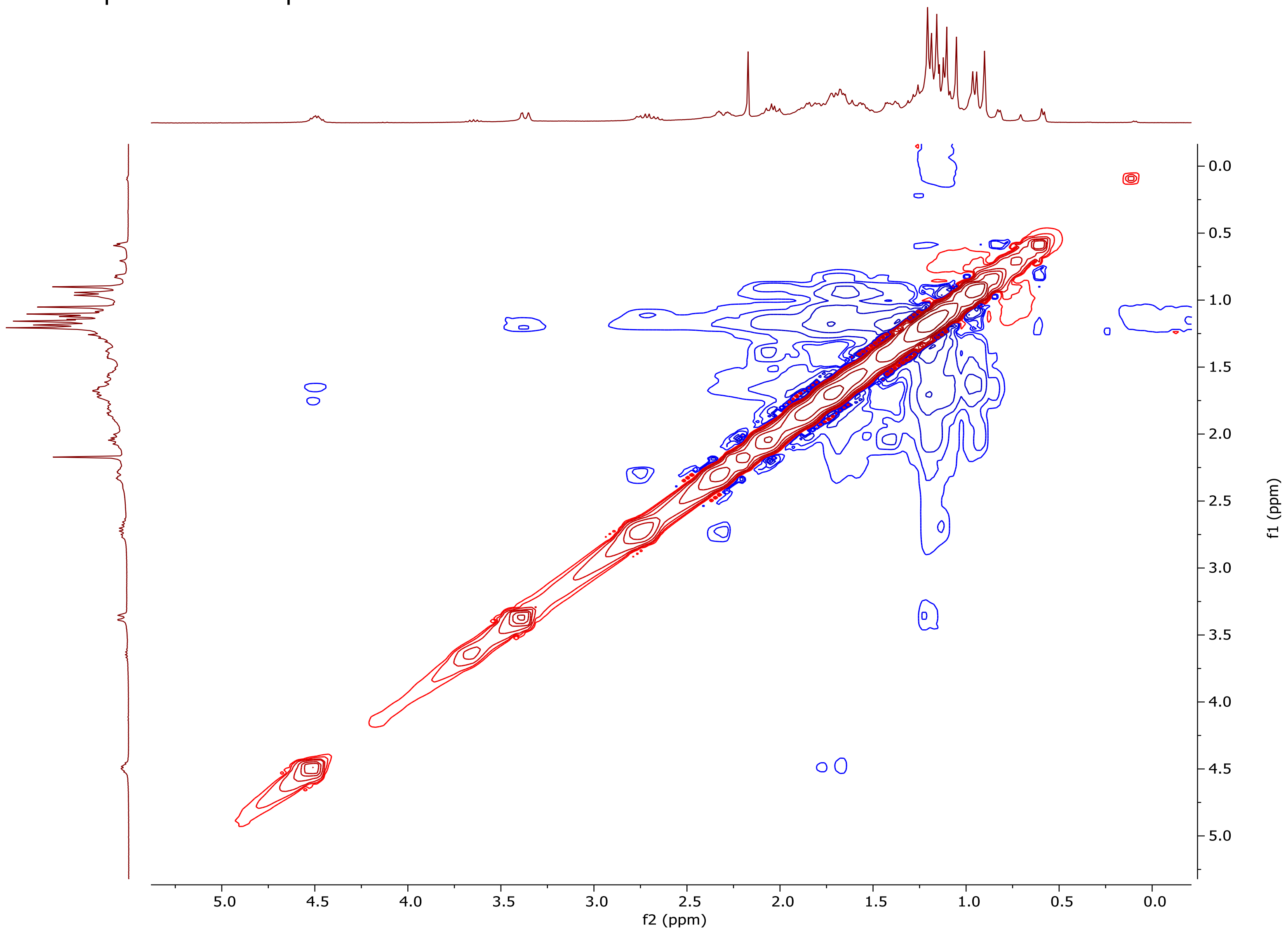

S9. HRMS spectrum of compound **7**.

Data:516 VD27pp
Sample Name:Dr Delgado Guillermo / Operador Javier Perez
Description:
Ionization Mode:ESI+
History:Determine m/z[Peak Detect[Centroid,30,Area];Correct Base[];Smooth[5]];Correct Base[5.0%];Average(MS[...

Acquired:3/2/2023 3:48:14 PM
Operator:AccuTOF
Mass Calibration data:Cal_PEG_600
Created:3/21/2023 2:34:25 PM
Created by:AccuTOF

Charge number:1
Element:^{12}C:0 .. 30, ^{1}H:0 .. 60, ^{16}O:0 .. 5

Tolerance:3.00(mmu)

Unsaturation Number:0.0 .. 50.0 (Fraction:Both)

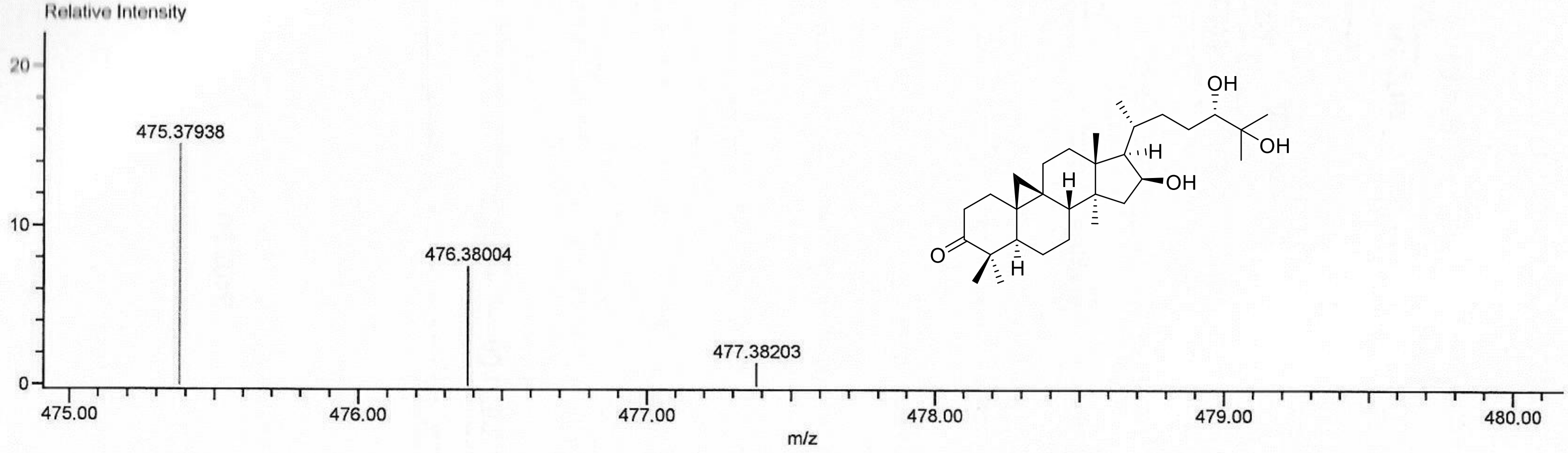

Mass	Intensity	Calc. Mass	Mass Difference (mmu)	Mass Difference (ppm)	Possible Formula	Unsaturation Number
475.37938	10930.47	475.37873	0.65	1.36	$^{12}C_{30}{}^{1}H_{51}{}^{16}O_{4}$	5.5

S10. IR spectrum of compound **8**.

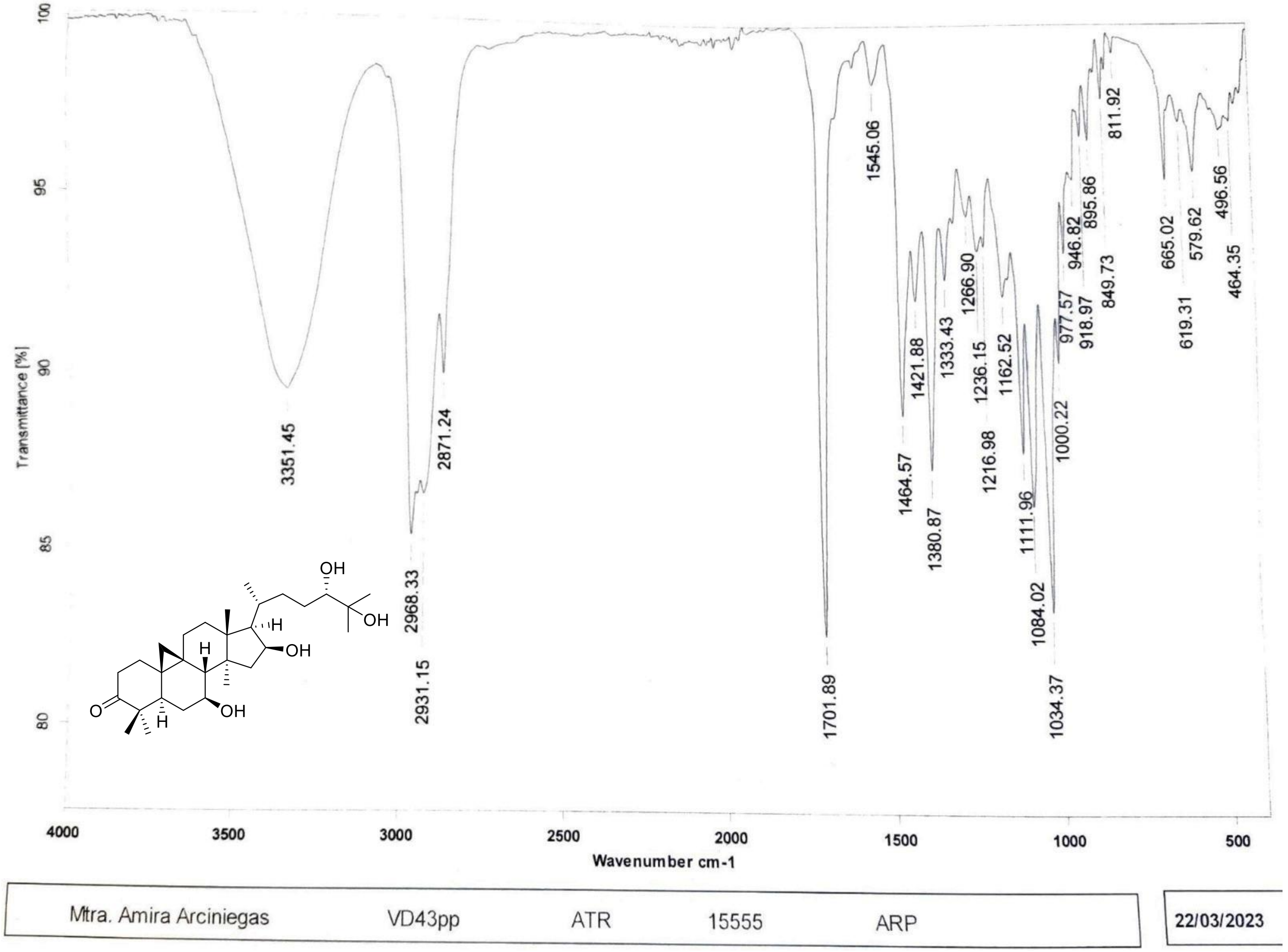

S11. ^{1}H NMR spectrum of compound **8**.

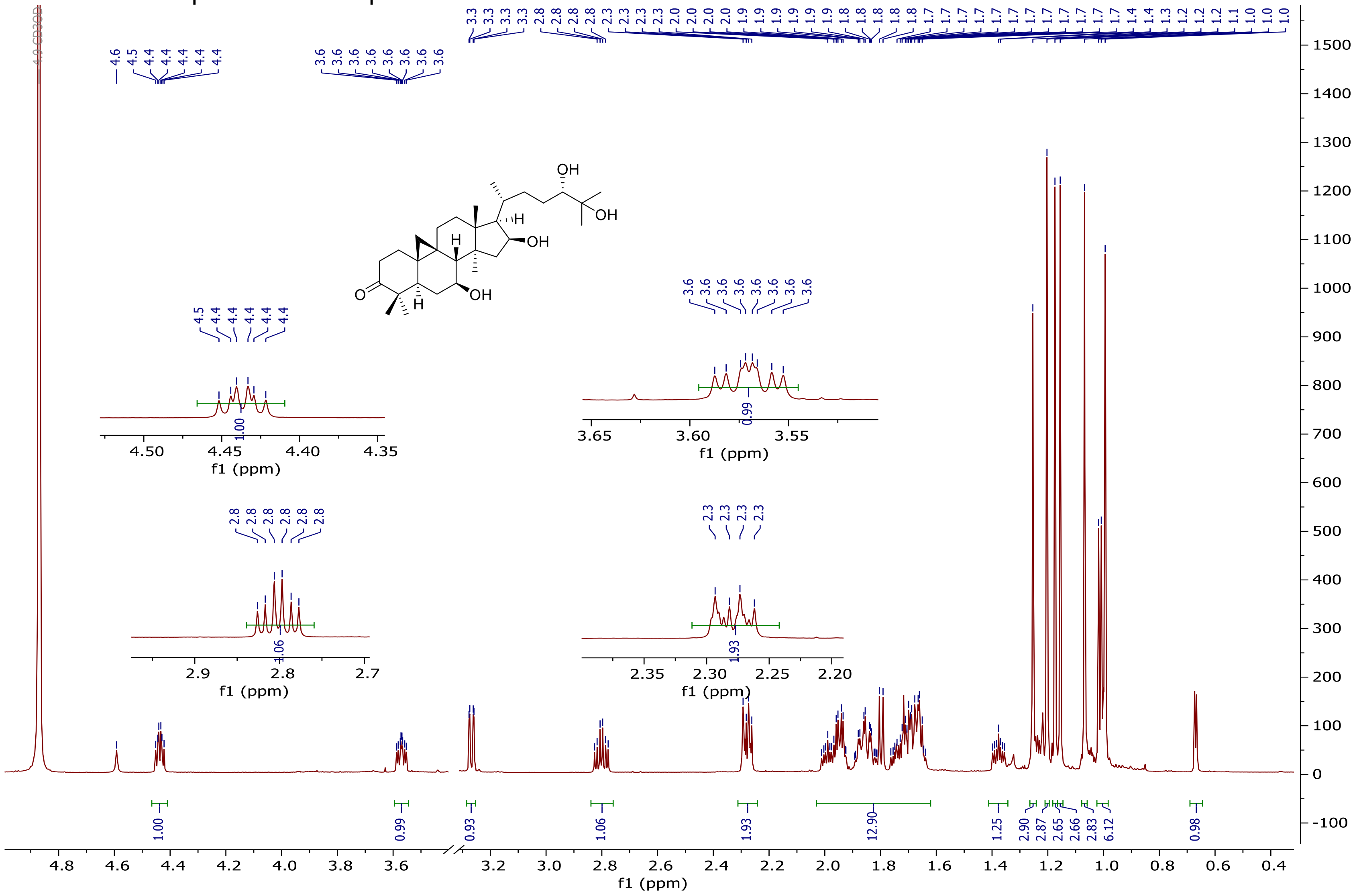

S12. ^{13}C NMR spectrum of compound **8**.

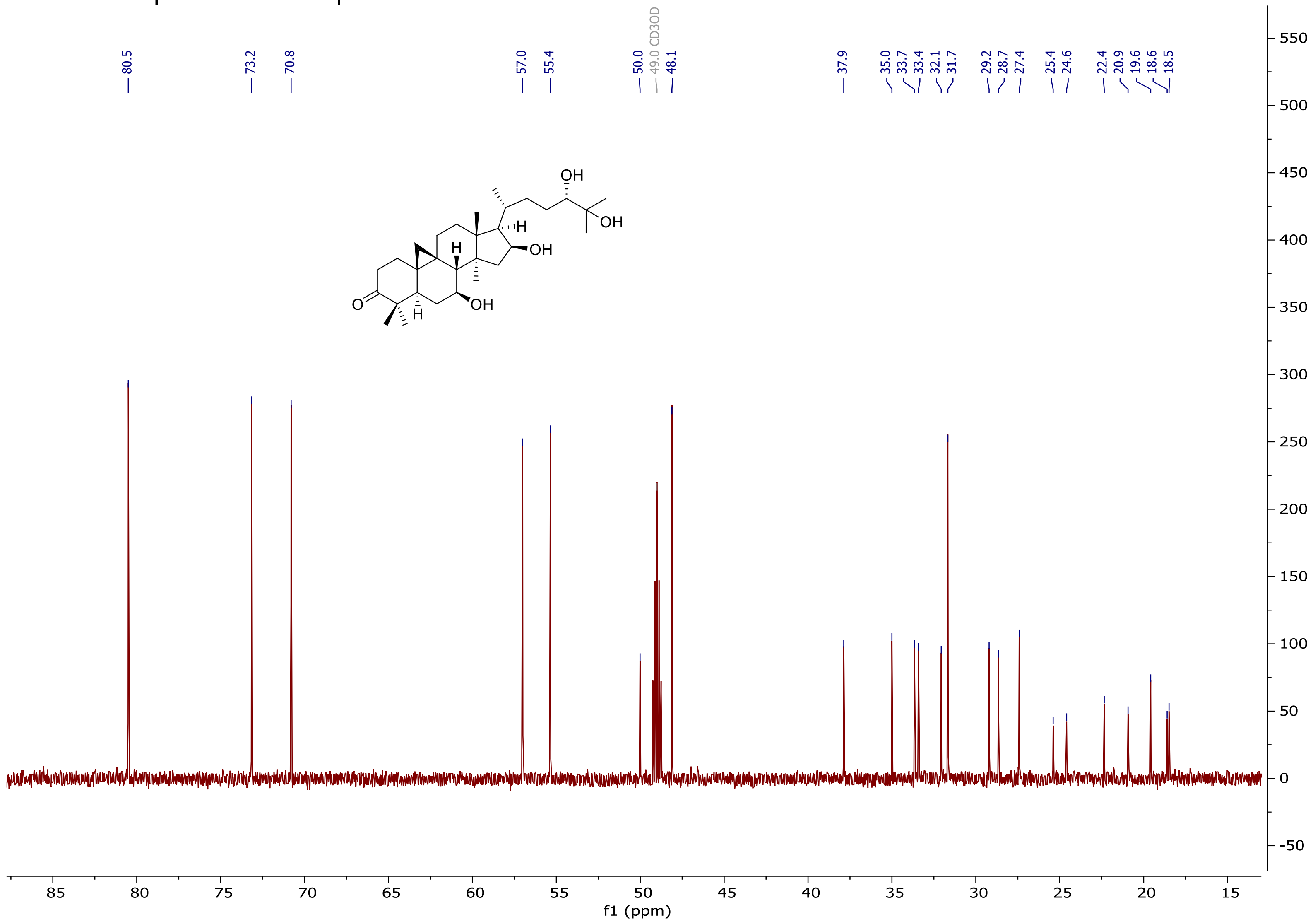

S13. COSY spectrum of compound **8**.

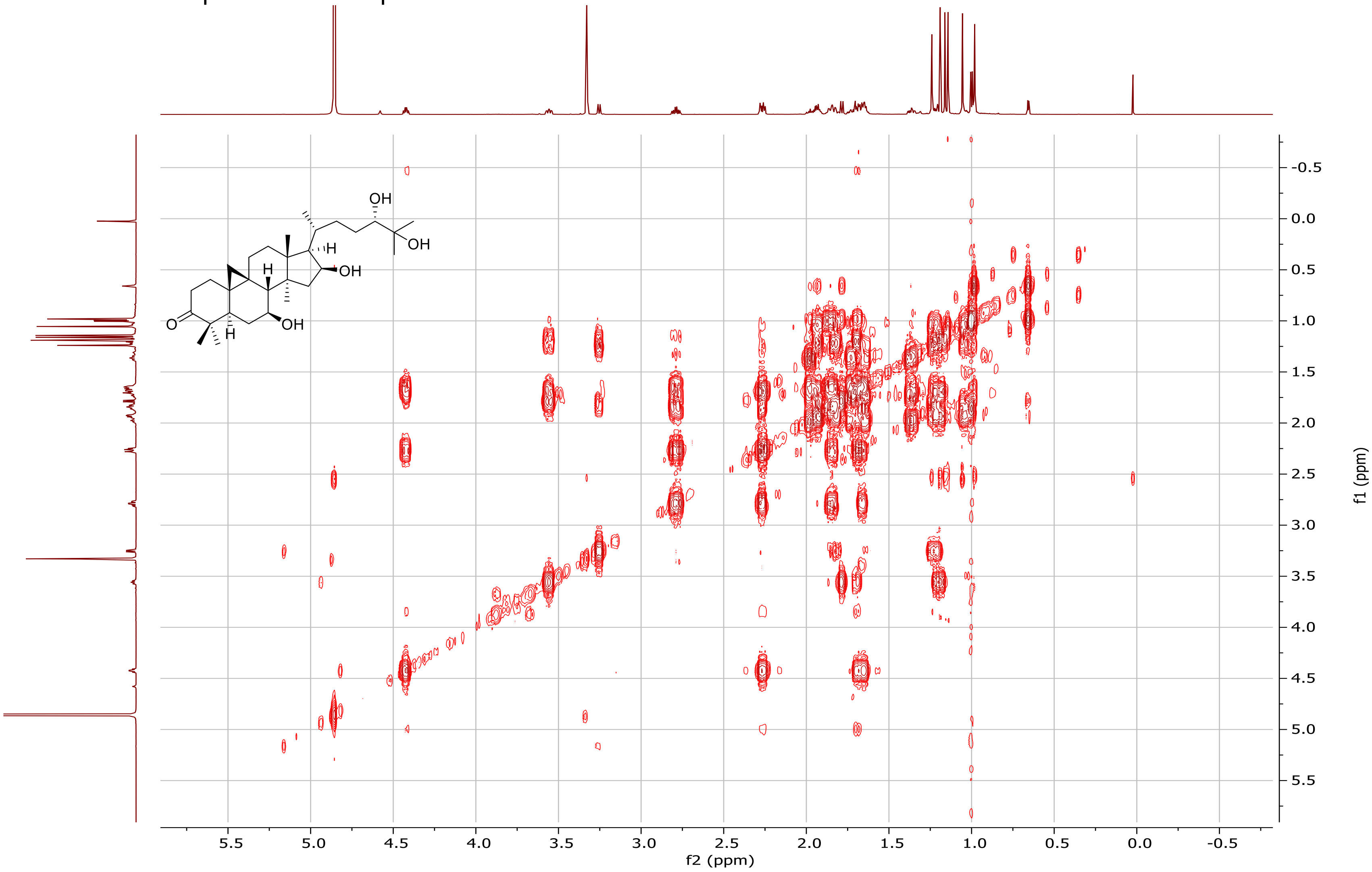

S14. HMBC spectrum of compound **8**.

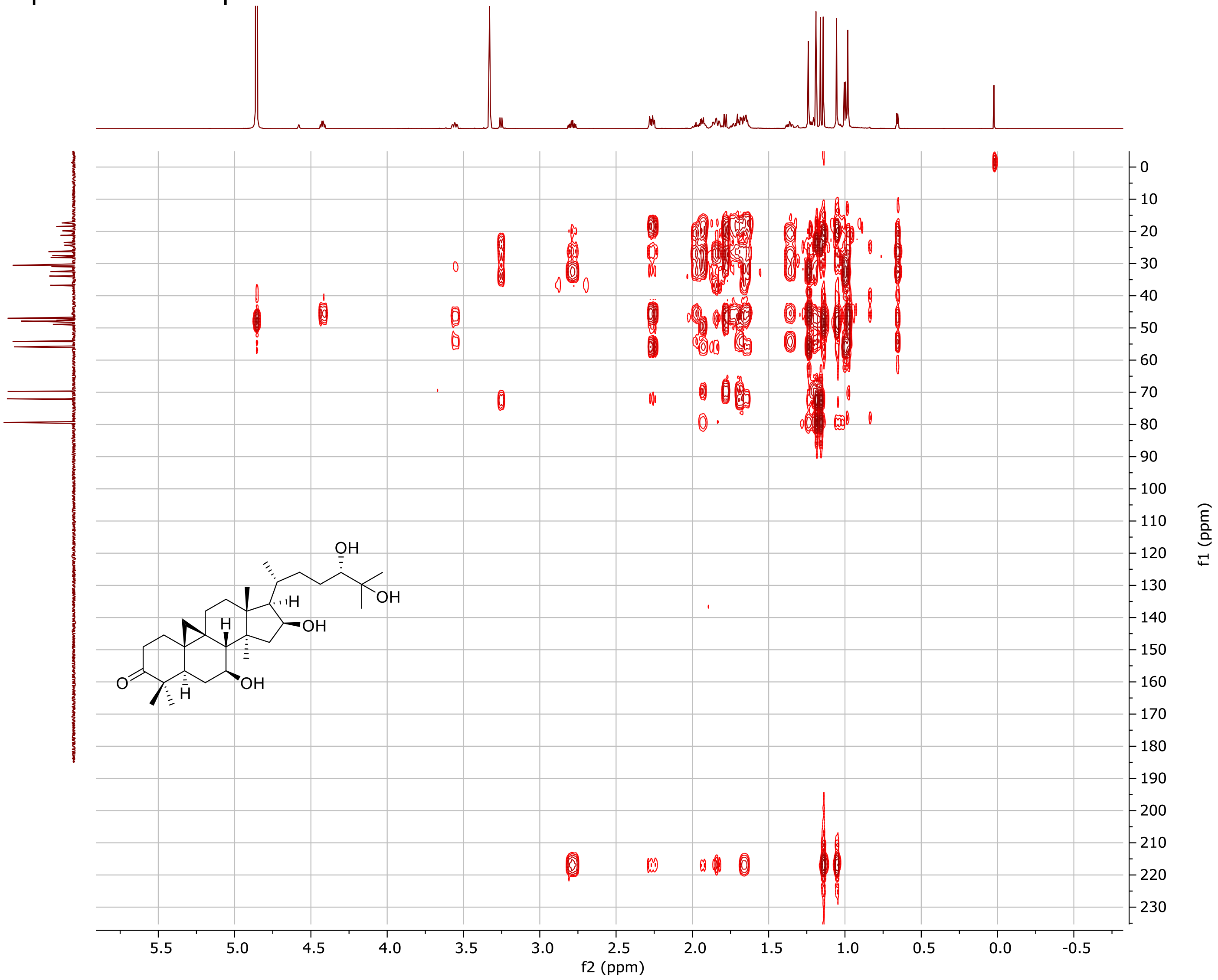

S17. HRMS spectrum of compound **8**.

Charge number:1

Element:^{12}C:0 .. 30, ^{1}H:0 .. 60, ^{16}O:0 .. 6

Tolerance:4.00(mmu)

Unsaturation Number:-1.0 .. 50.0 (Fraction:.5)

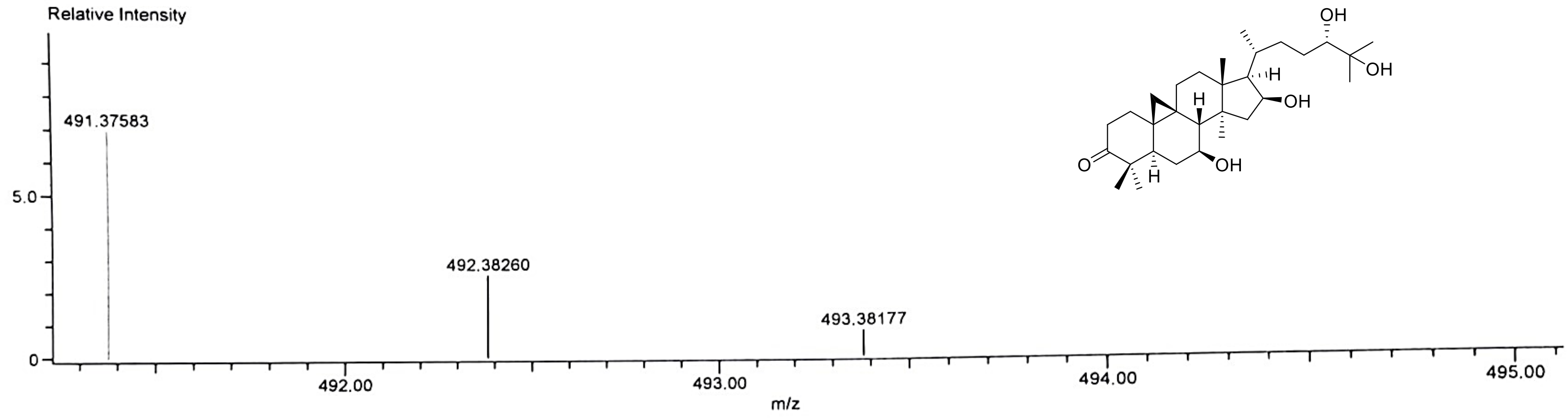

Mass	Intensity	Calc. Mass	Mass Difference (mmu)	Mass Difference (ppm)	Possible Formula	Unsaturation Number
491.37583	11161.30	491.37365	2.18	4.45	$^{12}C_{30}{}^{1}H_{51}{}^{16}O_5$	5.5

S18. IR spectrum of compound **9**.

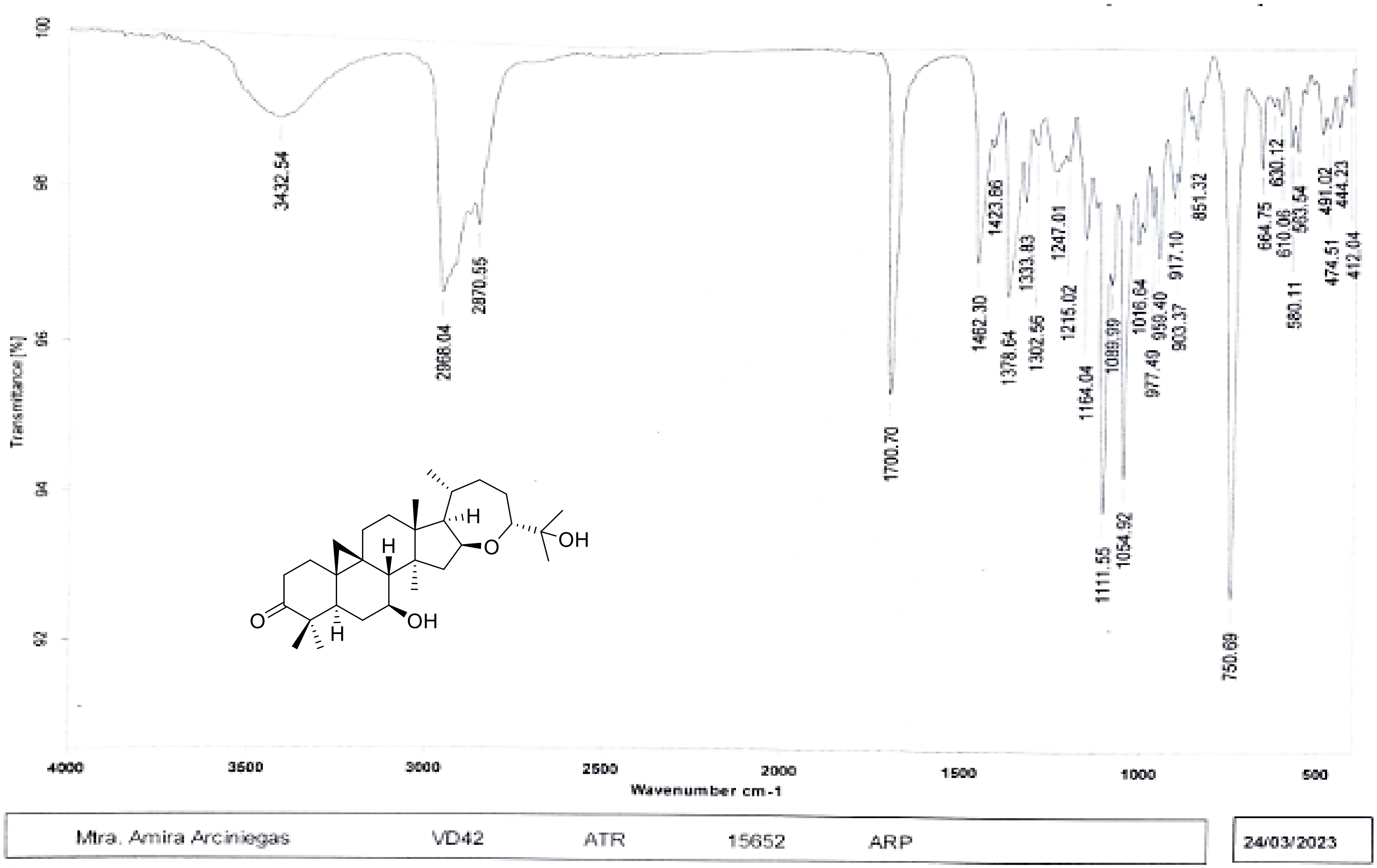

S19. ^{1}H NMR spectrum of compound **9**.

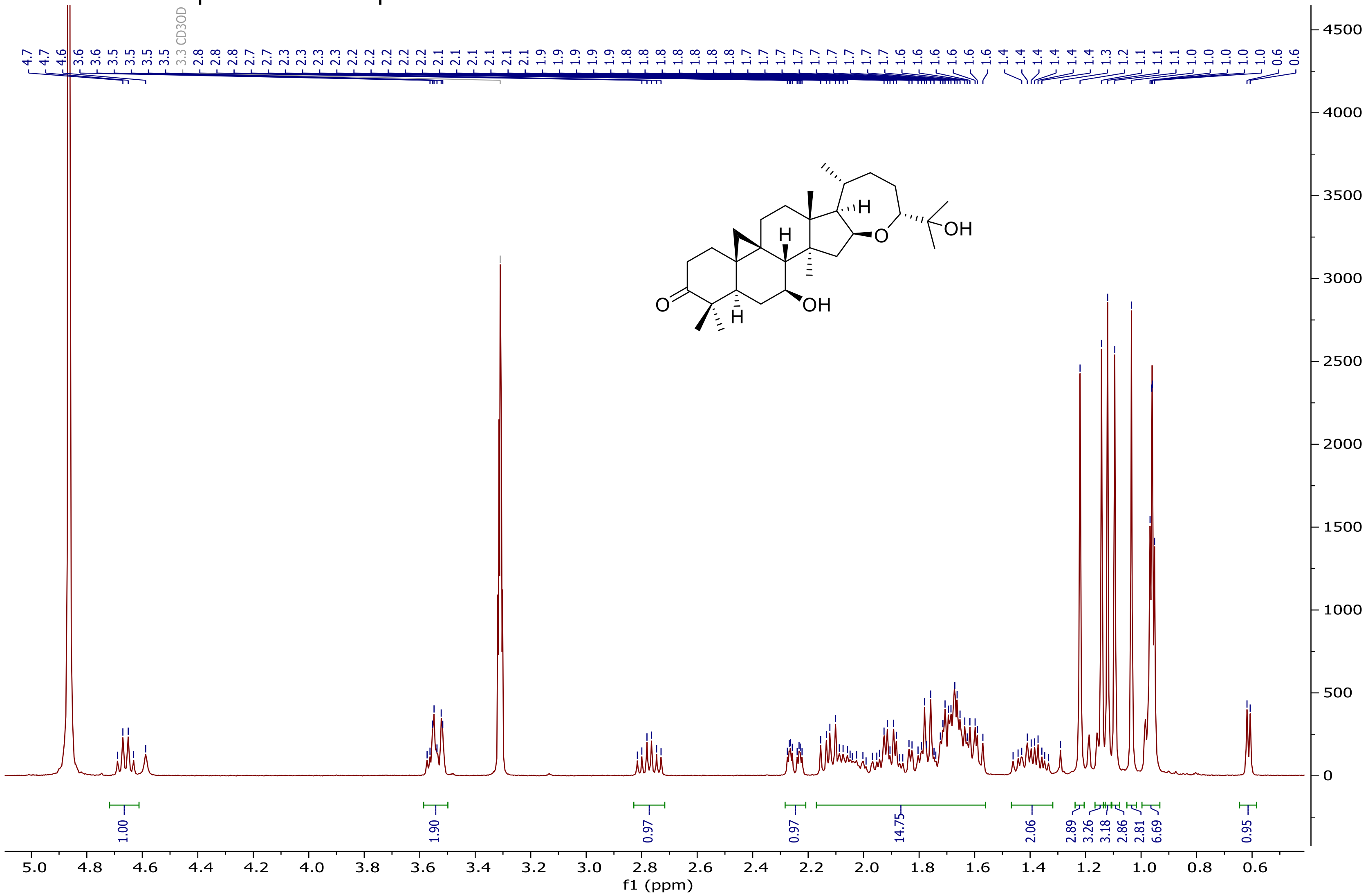

S20. ^{13}C NMR spectrum of compound **9**.

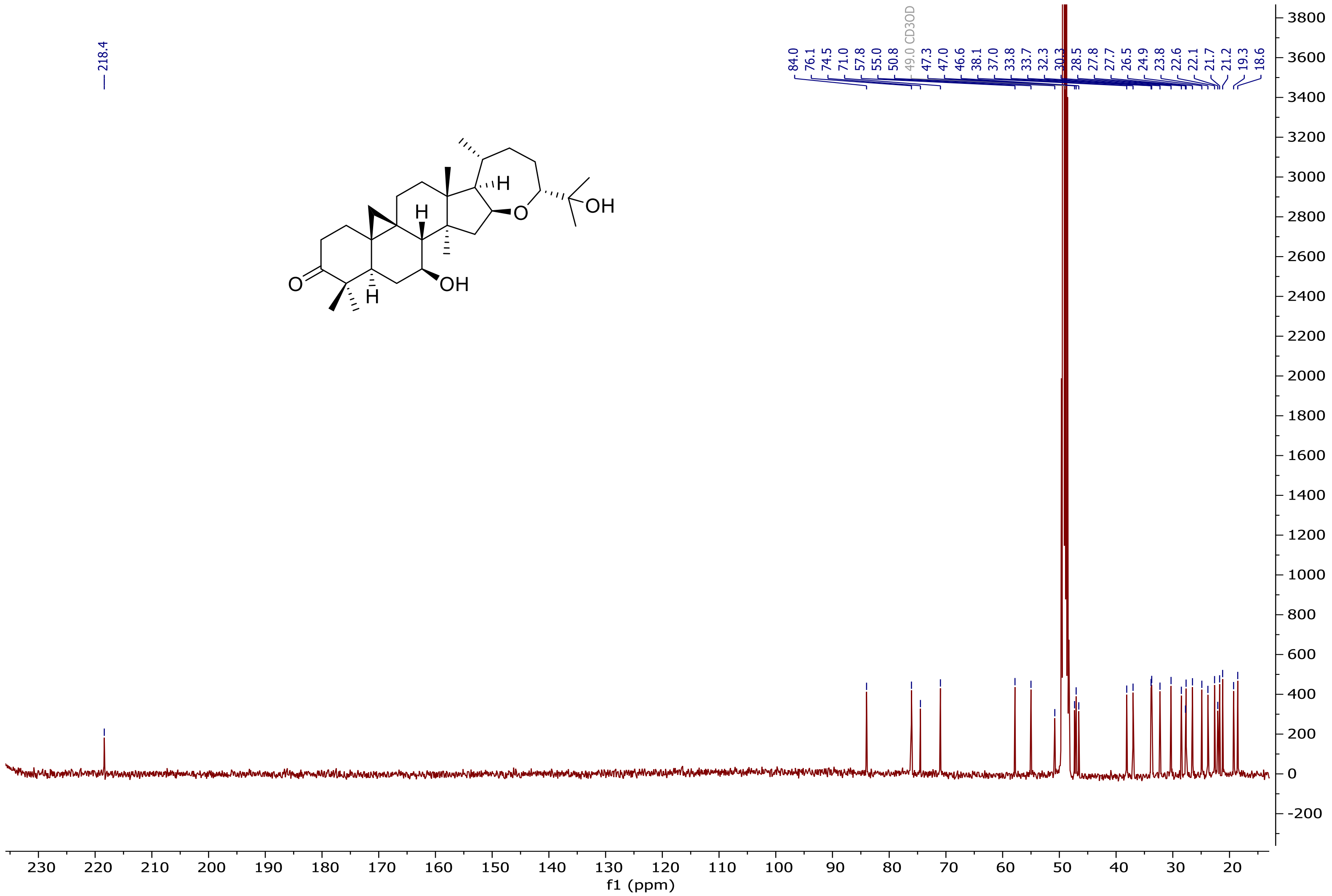

S21. COSY spectrum of compound **9**.

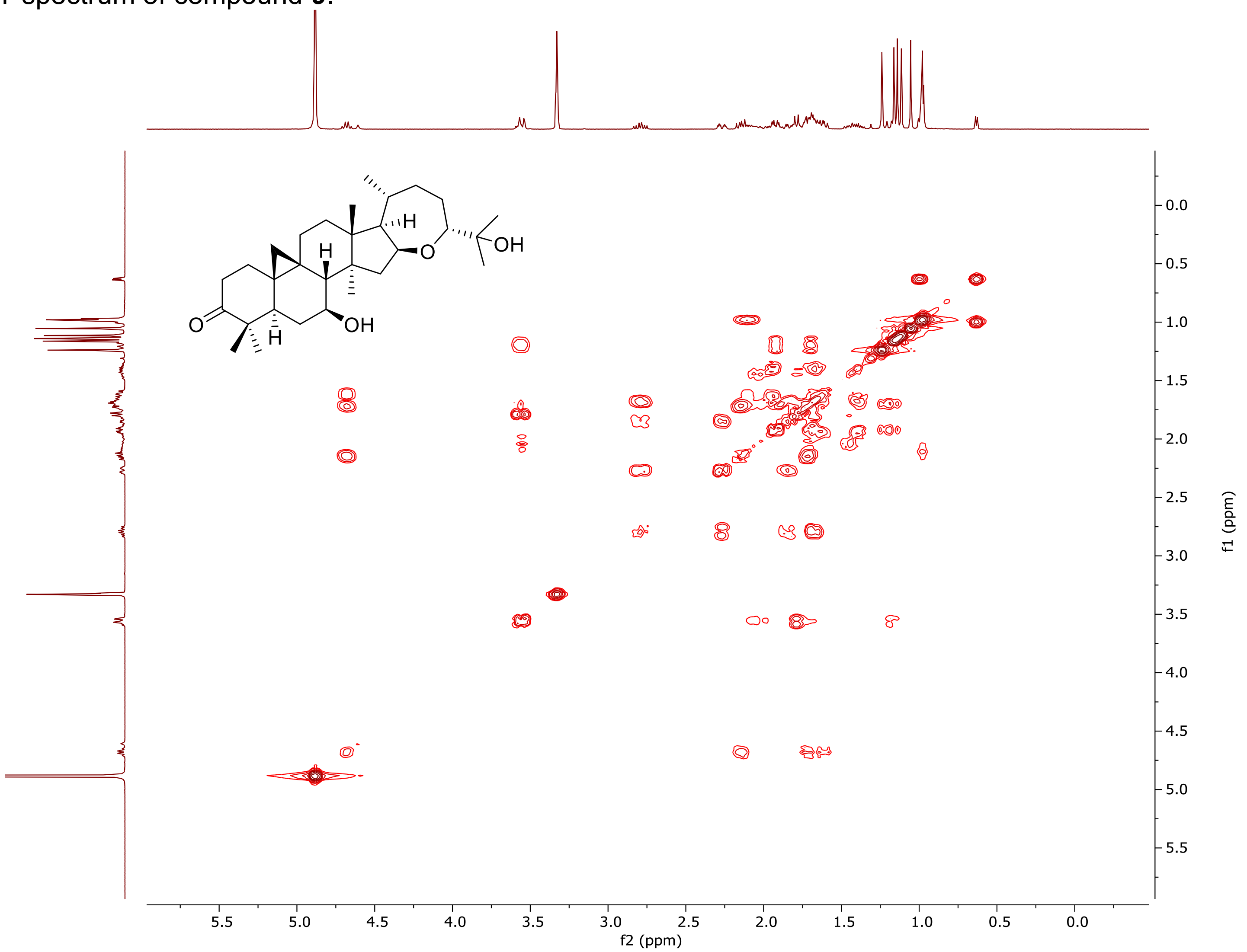

S22. HSQC spectrum of compound **9**.

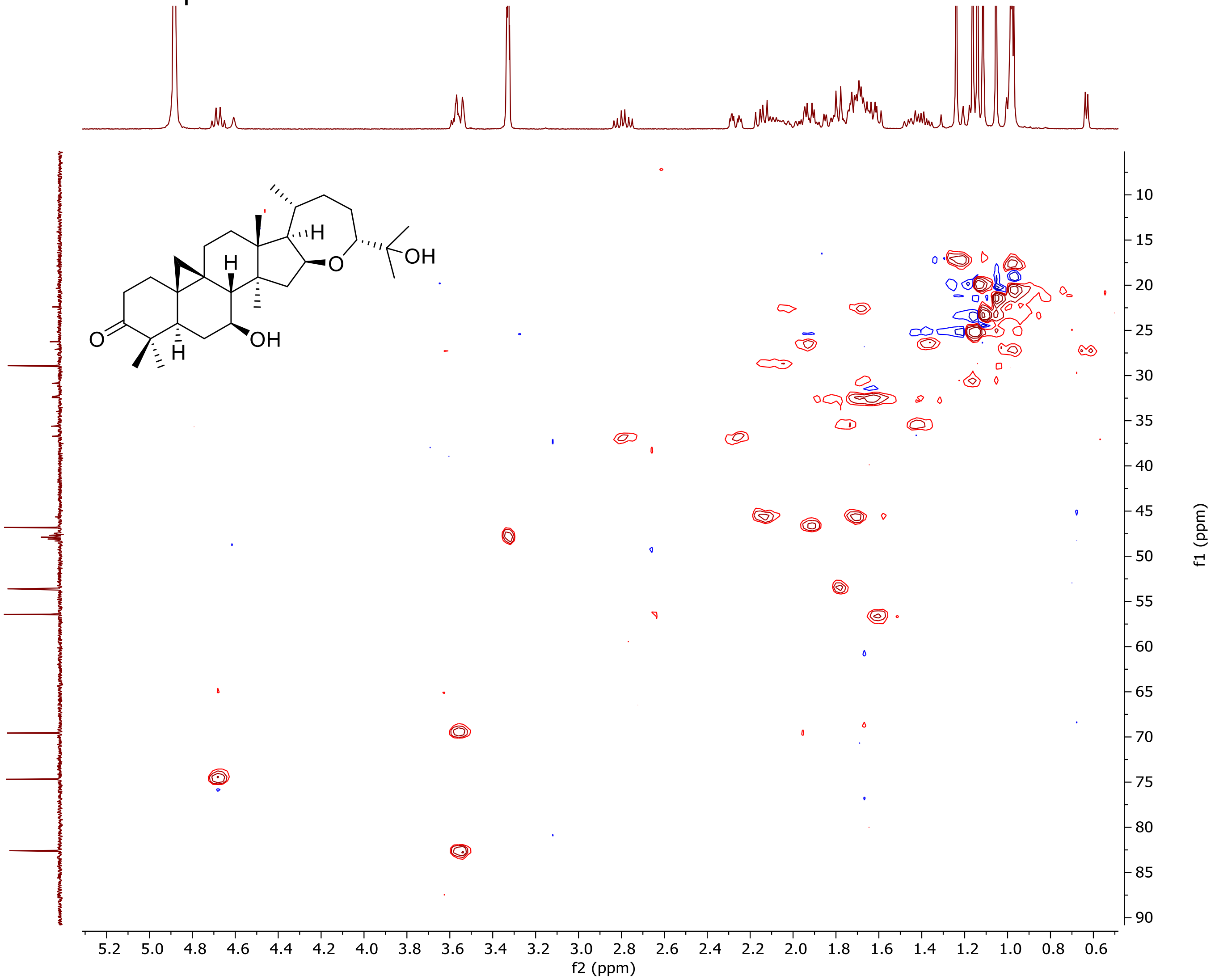

S23. HMBC spectrum of compound **9**.

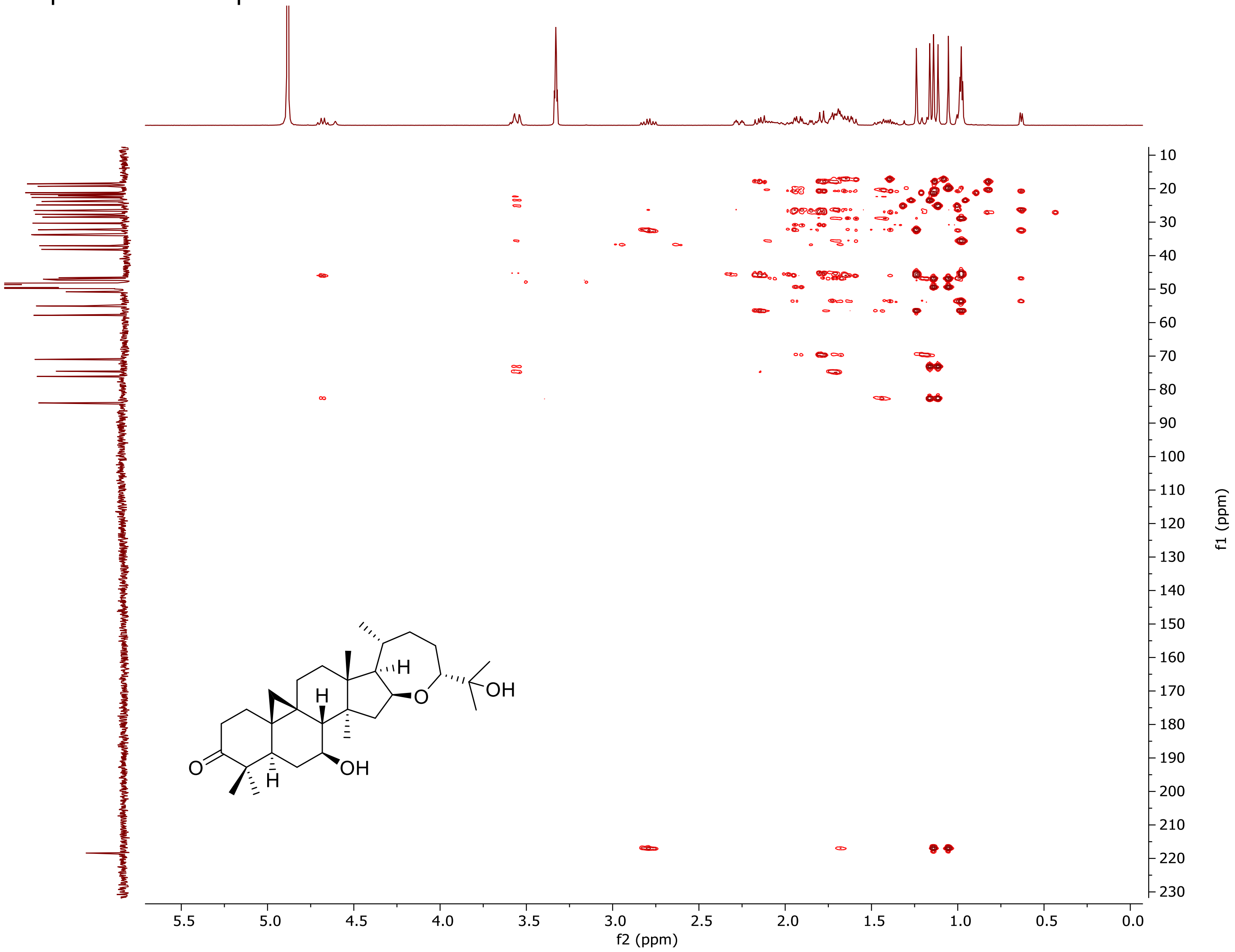

S24. NOESY spectrum of compound **9**.

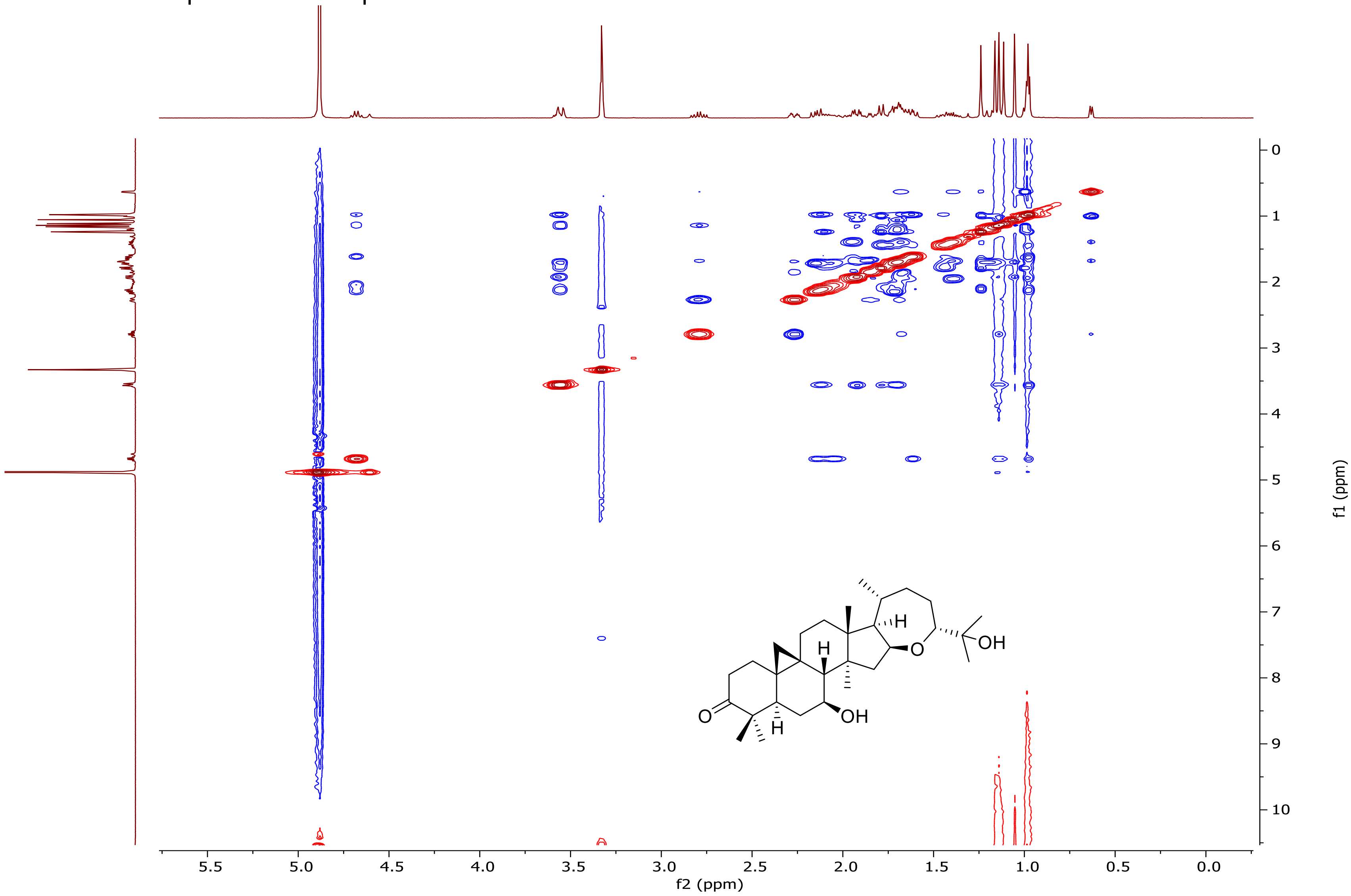

S25. HRMS spectrum of compound **9**.

INSTITUTO DE QUIMICA, UNAM
LABORATORIO DE ESPECTROMETRIA DE MASAS

Data:1949_VD42
Sample Name:Dr Delgado Guillermo / Operador: Carmen Garcia
Description:
Ionization Mode:ESI+
History:Determine m/z[Peak Detect[Centroid,30,Area];Correct Base[];Smooth[5]];Correct Base[5.0%];Average(MS[...

Acquired:7/25/2022 10:43:43 AM
Operator:AccuTOF
Mass Calibration data:Cal_PEG_600
Created:9/7/2022 12:34:33 PM
Created by:AccuTOF

Charge number:1
Element:^{12}C:0 .. 50, ^{1}H:0 .. 60, ^{16}O:0 .. 6

Tolerance:10.00(mmu)

Unsaturation Number:0.0 .. 50.0 (Fraction:.5)

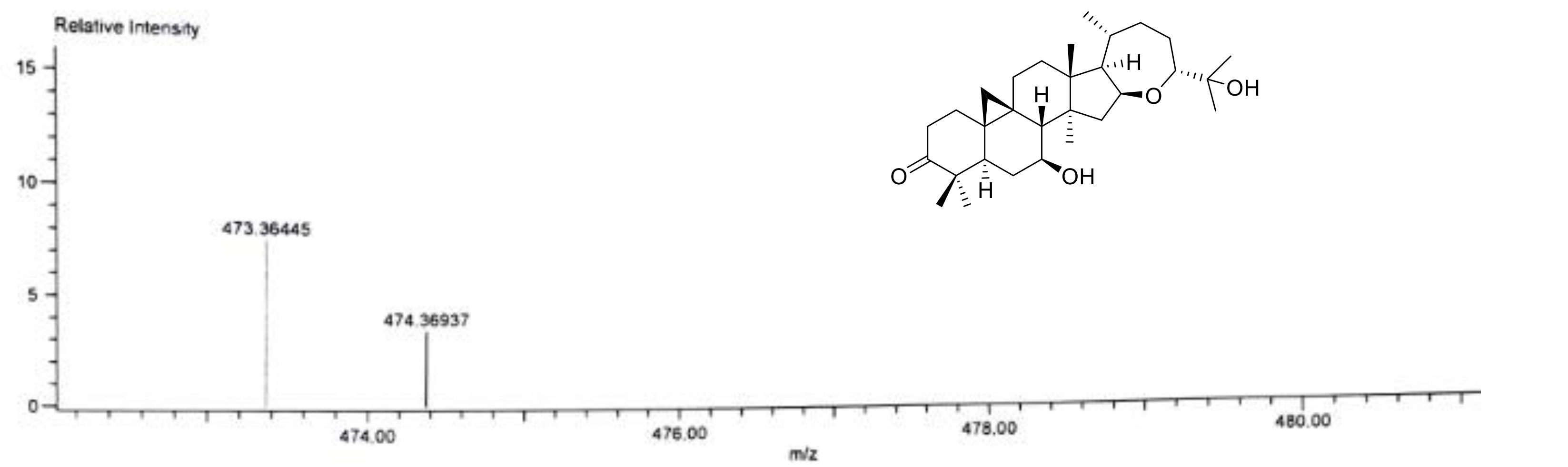

Mass	Intensity	Calc. Mass	Mass Difference (mmu)	Mass Difference (ppm)	Possible Formula	Unsaturation Number
473.36445	1043.08	473.36308	1.37	2.90	$^{12}C_{30}{}^{1}H_{49}{}^{16}O_{4}$	6.5

Crystal Data and Structure Refinement of compound **9**.

Table 1. Crystal data and structure refinement for **VD-42**.

Identification code	038AAA23	
Empirical formula	$C_{30}H_{48}O_4$	
Formula weight	472.68	
Temperature	373(2) K	
Wavelength	0.71073 Å	
Crystal system	Monoclinic	
Space group	$P2_1$	
Unit cell dimensions	a = 15.673(4) Å	α= 90°.
	b = 6.0278(11) Å	β= 117.56(3)°.
	c = 15.800(4) Å	γ = 90°.
Volume	1323.2(6) $Å^3$	
Z	2	
Density (calculated)	1.186 Mg/m^3	
Absorption coefficient	0.076 mm^{-1}	
F(000)	520	
Crystal size	0.3560 x 0.1942 x 0.1406 mm^3	
Theta range for data collection	3.680 to 28.576°.	
Index ranges	-21<=h<=20, -7<=k<=7, -20<=l<=20	
Reflections collected	9441	
Independent reflections	5333 [R(int) = 0.0718]	
Completeness to theta = 25.242°	99.6 %	
Absorption correction	Semi-empirical from equivalents	
Max. and min. transmission	1.00000 and 0.71255	
Refinement method	Full-matrix least-squares on F^2	
Data / restraints / parameters	5333 / 3 / 320	
Goodness-of-fit on F^2	1.043	
Final R indices [I>2sigma(I)]	R1 = 0.0762, wR2 = 0.1611	
R indices (all data)	R1 = 0.1145, wR2 = 0.1907	
Absolute structure parameter	1.5(10)	
Extinction coefficient	n/a	
Largest diff. peak and hole	0.294 and -0.422 e.$Å^{-3}$	

S26. ^{1}H NMR spectrum of compound **17**.

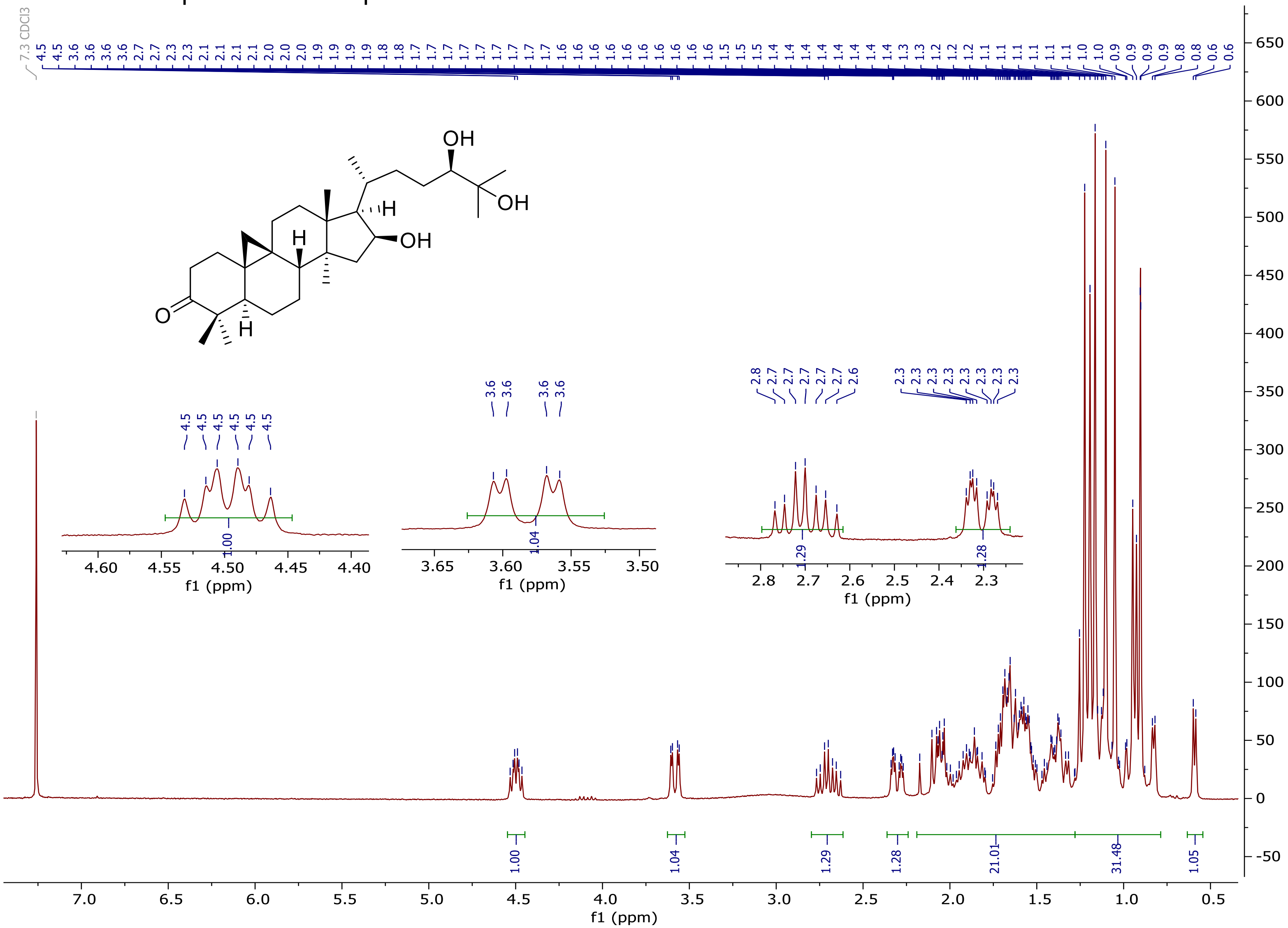

S27. ^{13}C NMR spectrum of compound **17**.

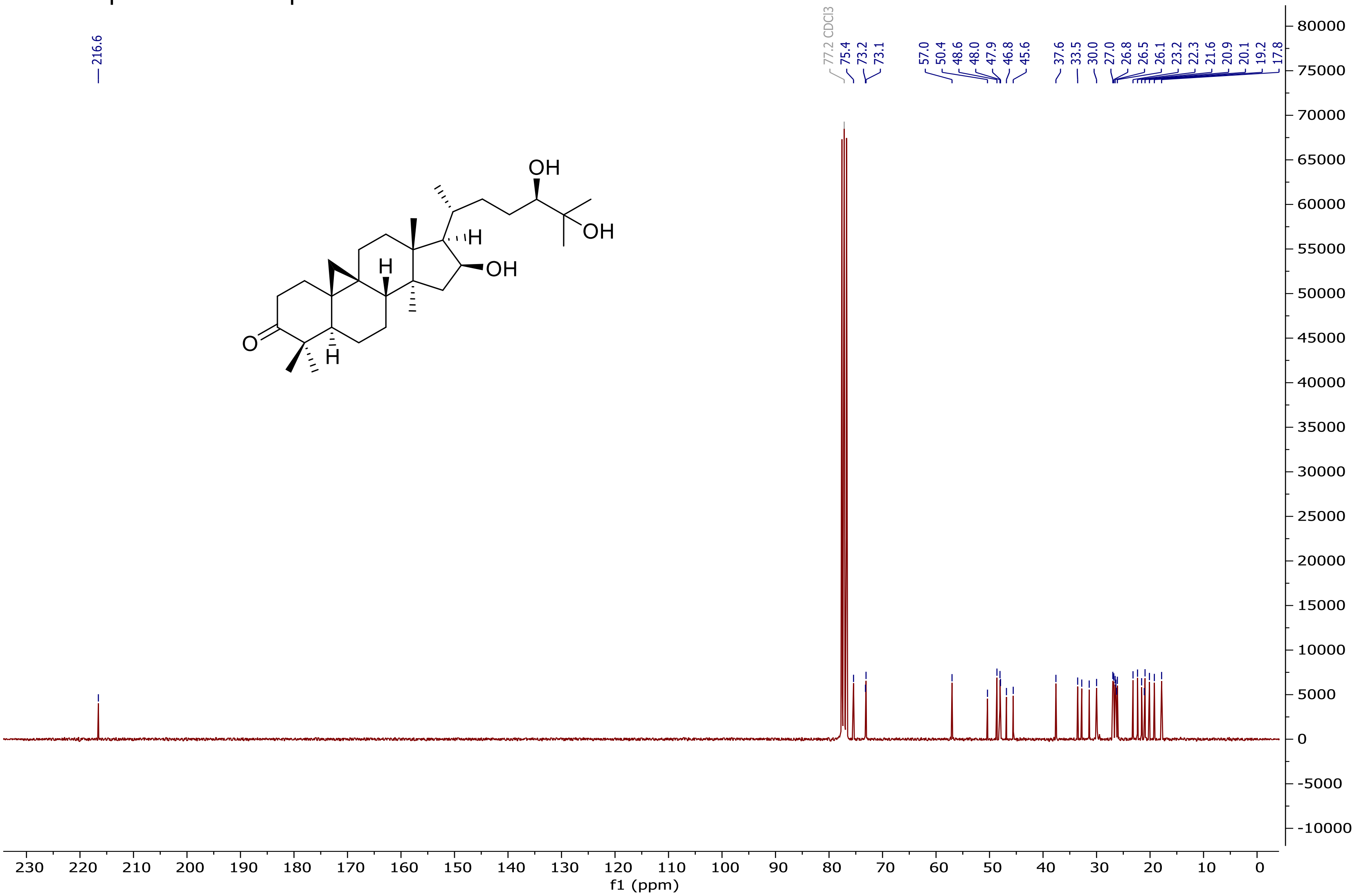

S28. COSY spectrum of compound **17**.

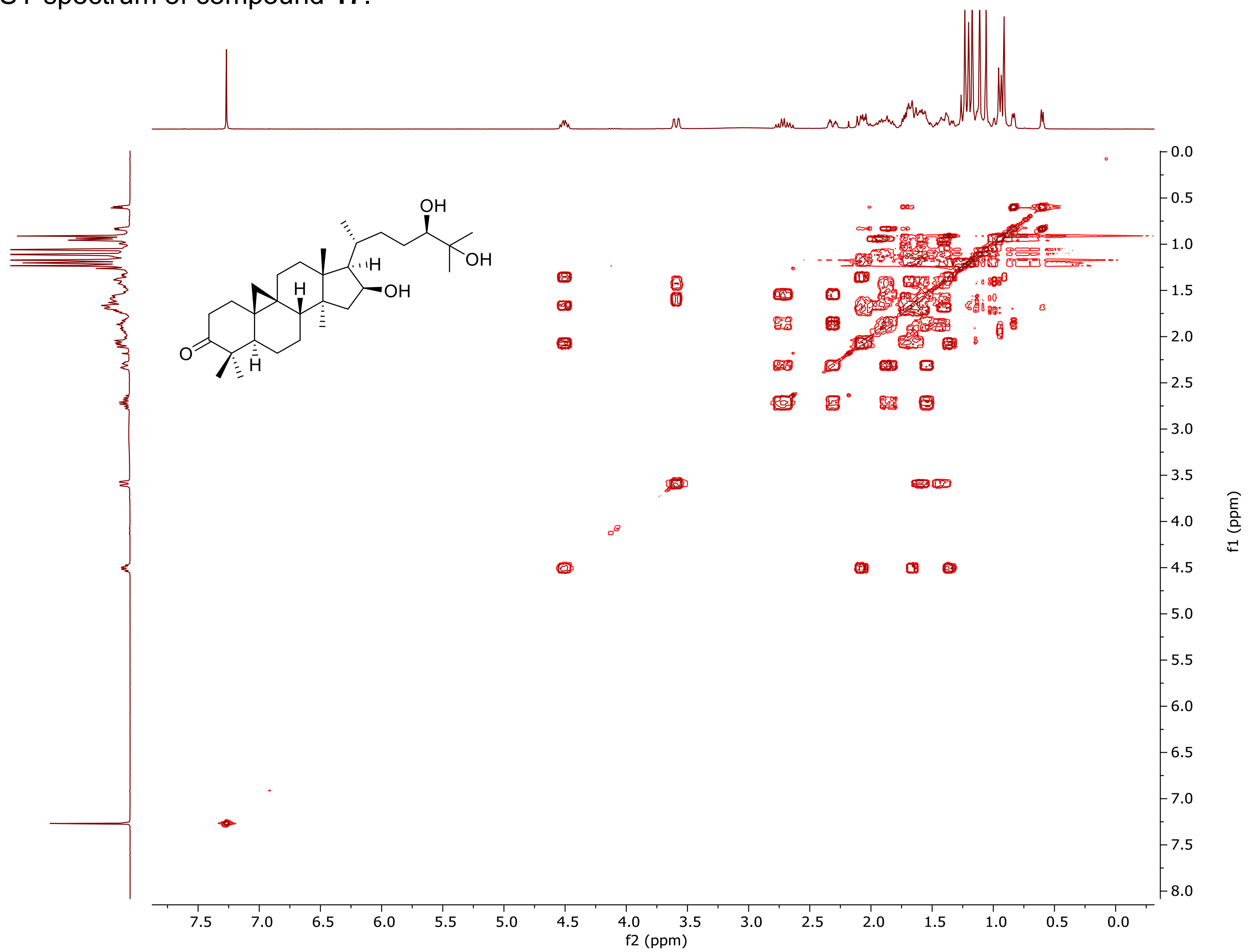

S29. HSQC spectrum of compound **17**.

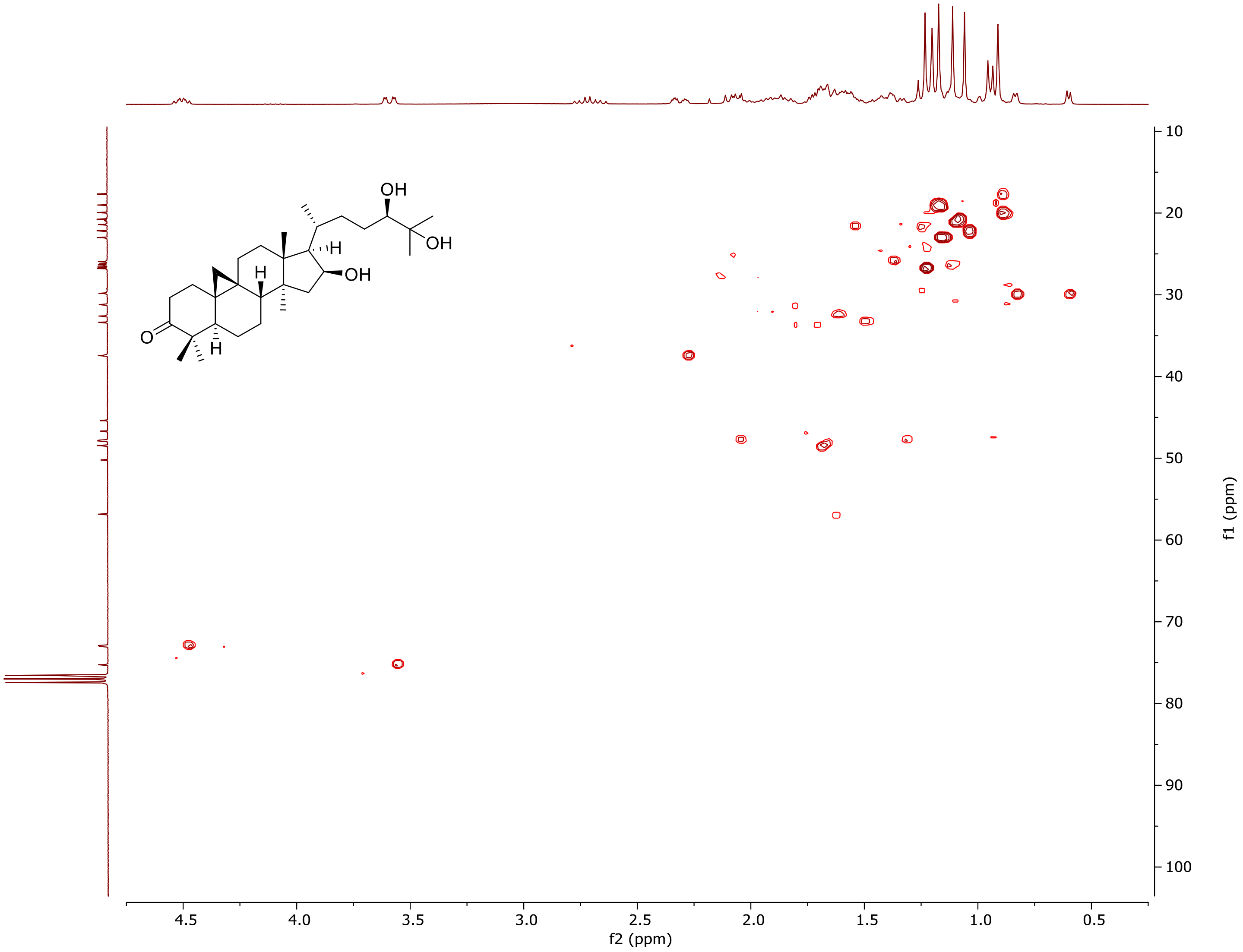

S30. HMBC spectrum of compound **17**.

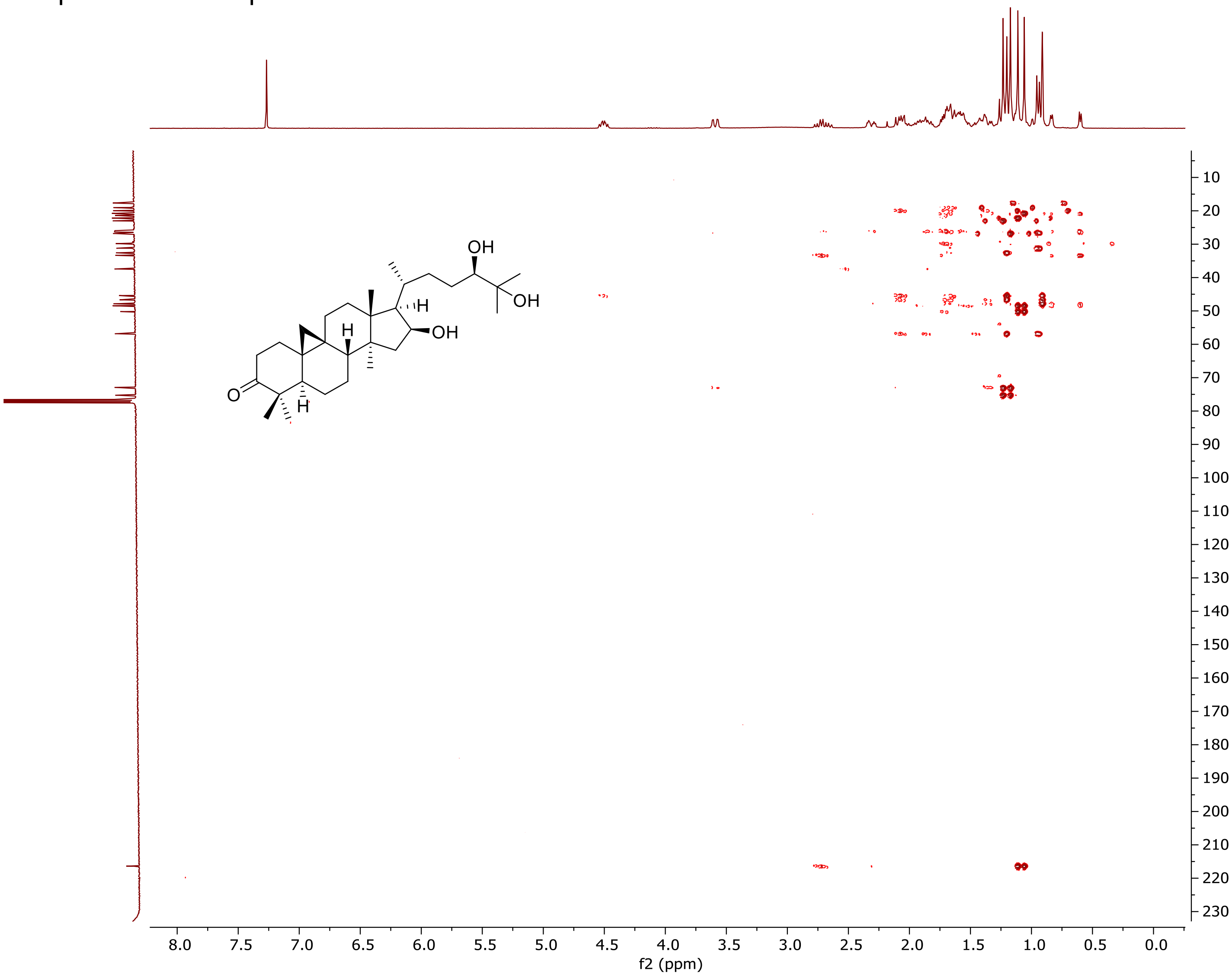

S31. HMBC expansion of compound **17**.

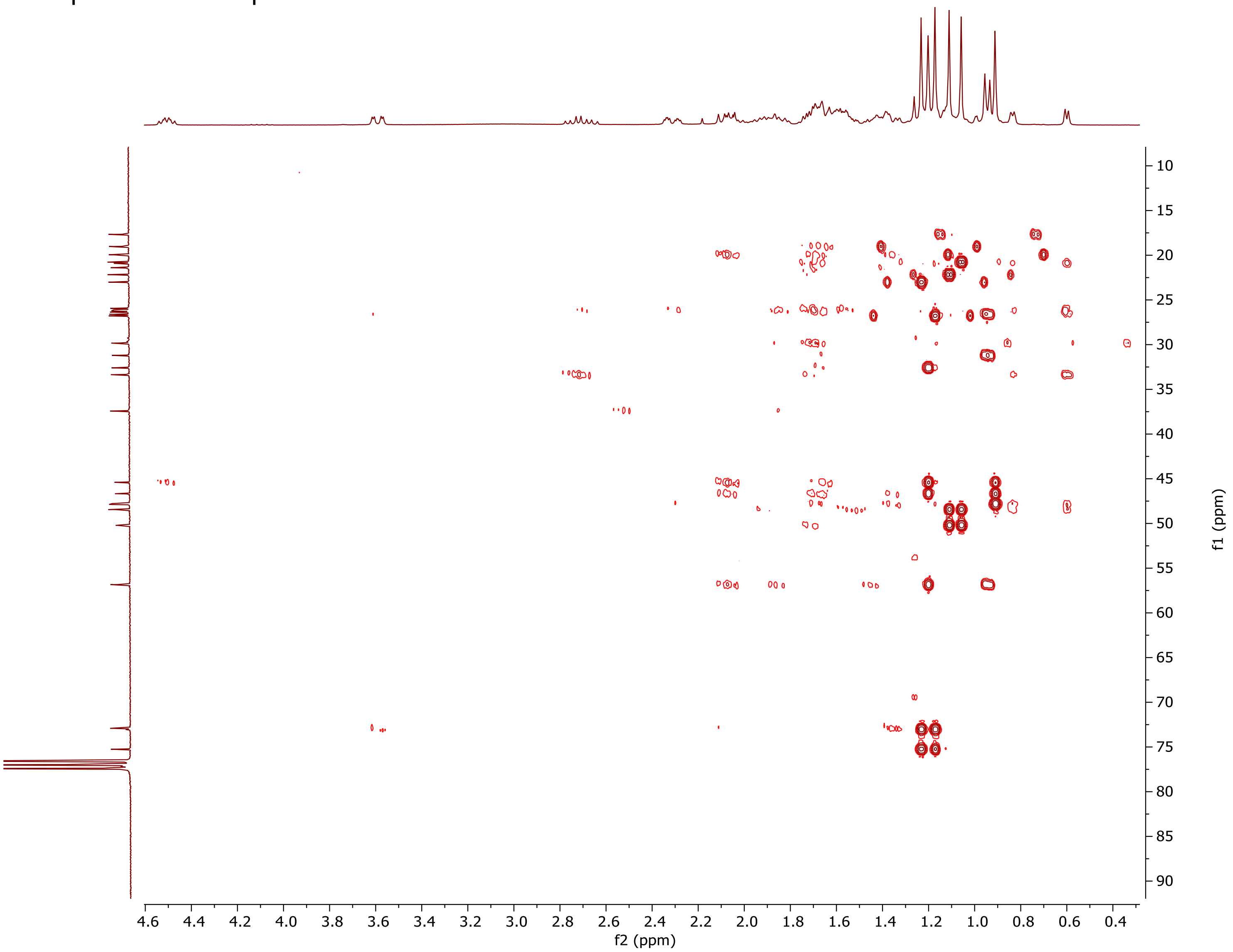

S32. NOESY spectrum of compound **17**.

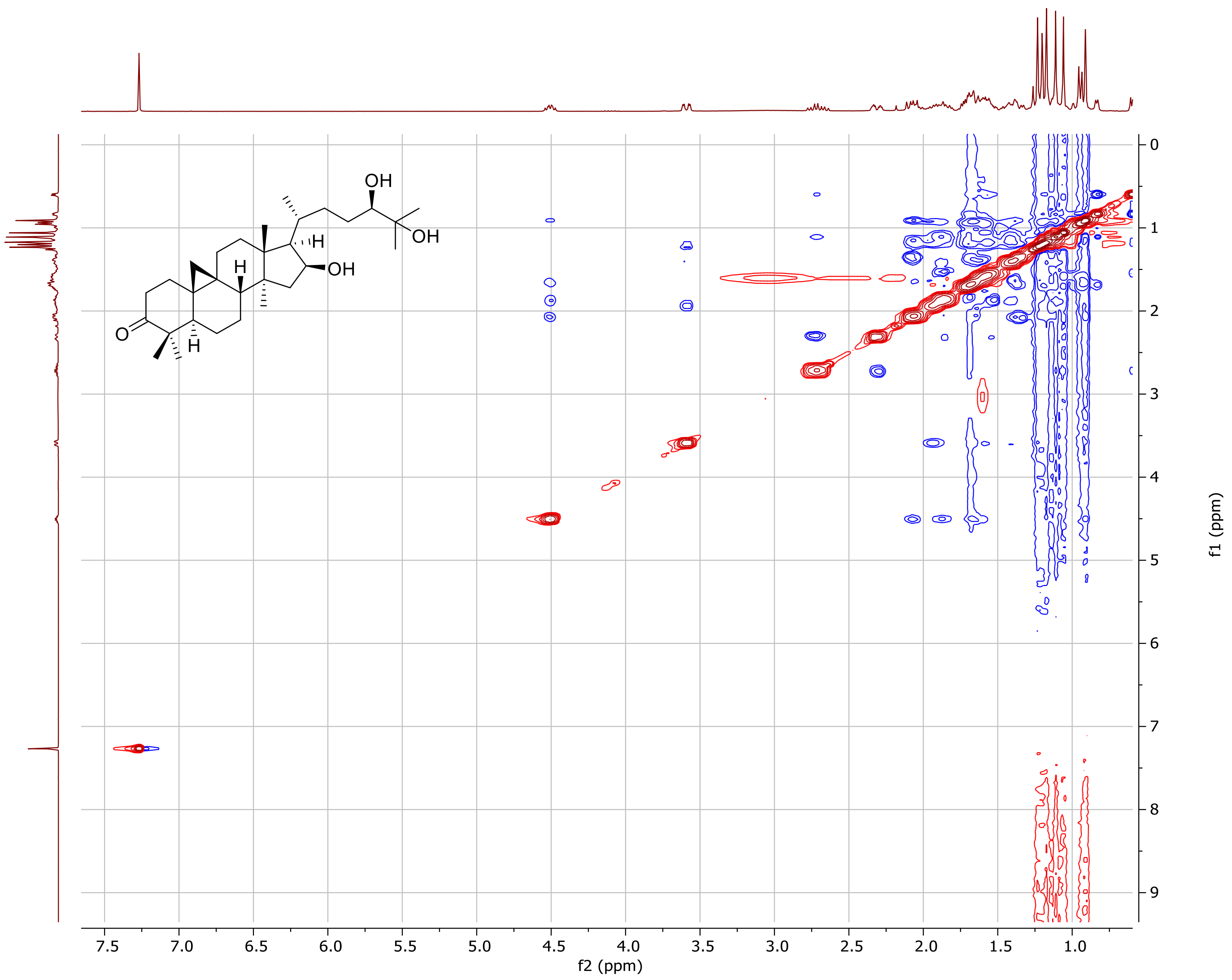

2. Molecular docking

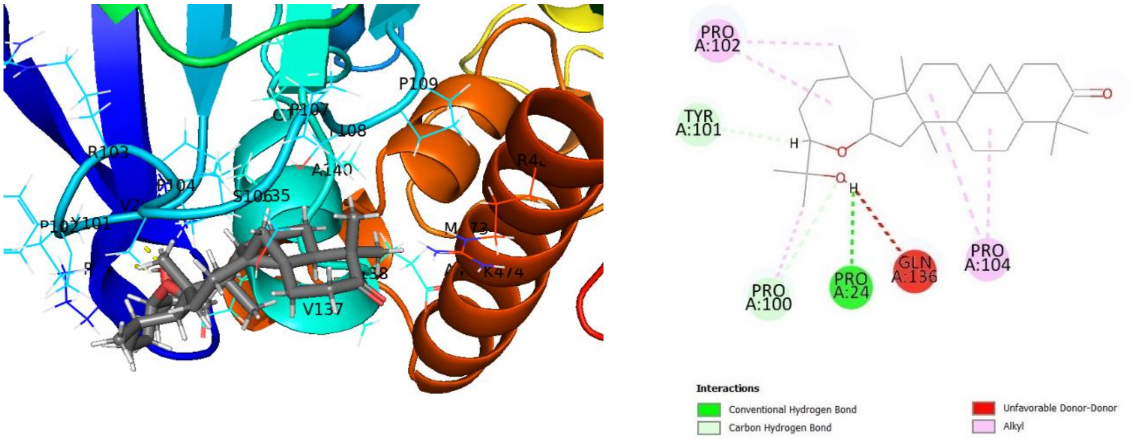

Figure S1. Molecular docking of compound **1** with acetylcholinesterase.

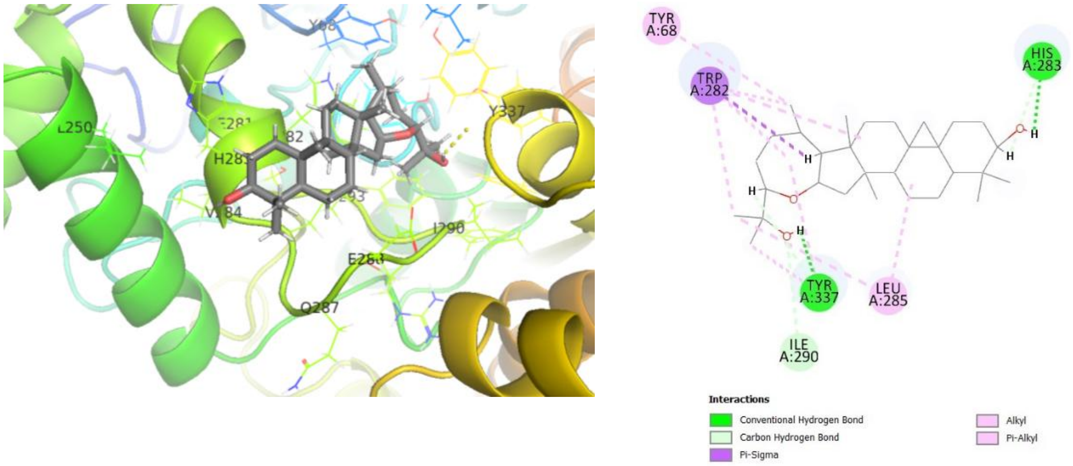

Figure S2. Molecular docking of compound **2** with acetylcholinesterase.

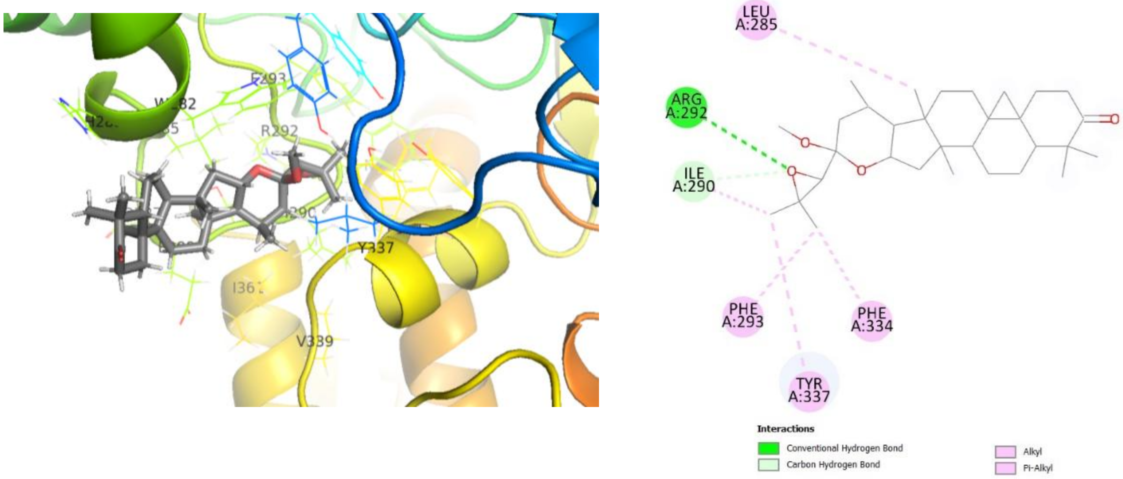

Figure S3. Molecular docking of compound **3** with acetylcholinesterase.

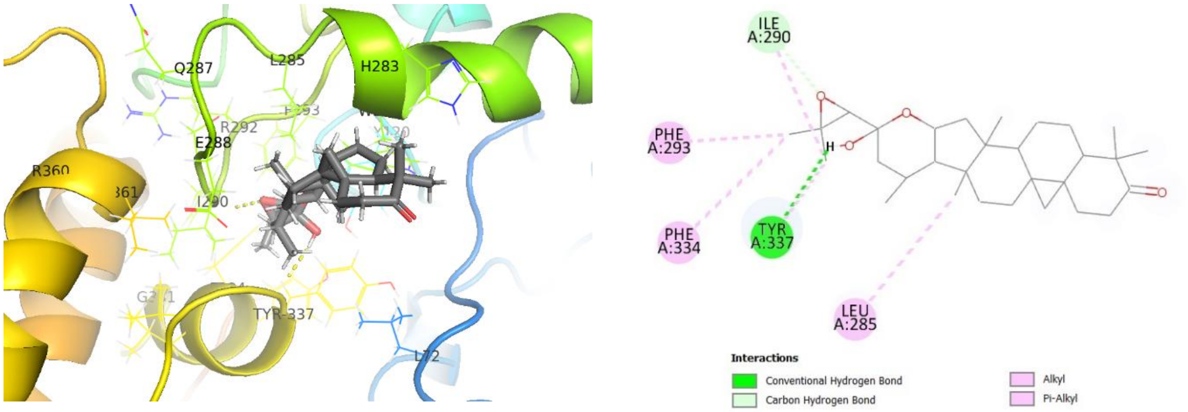

Figure S4. Molecular docking of compound **4** with acetylcholinesterase.

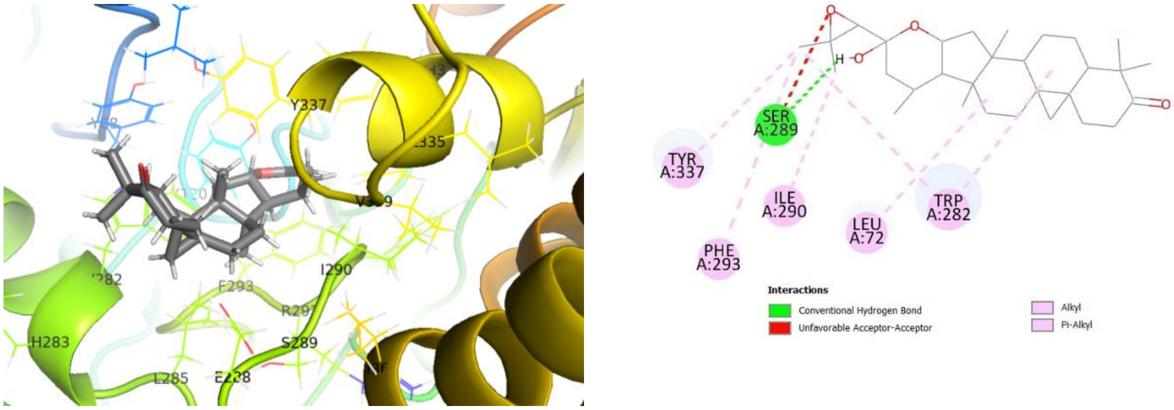

Figure S5. Molecular docking of compound **5** with acetylcholinesterase.

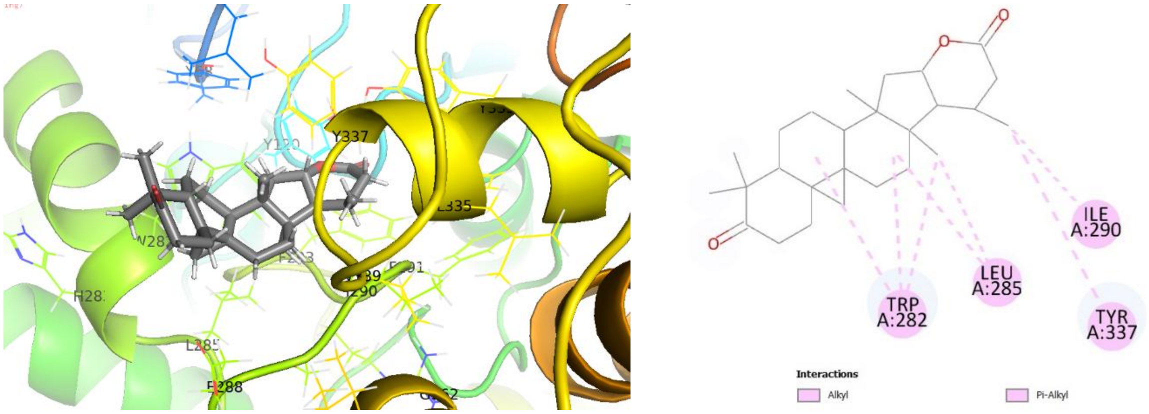

Figure S6. Molecular docking of compound **6** with acetylcholinesterase.

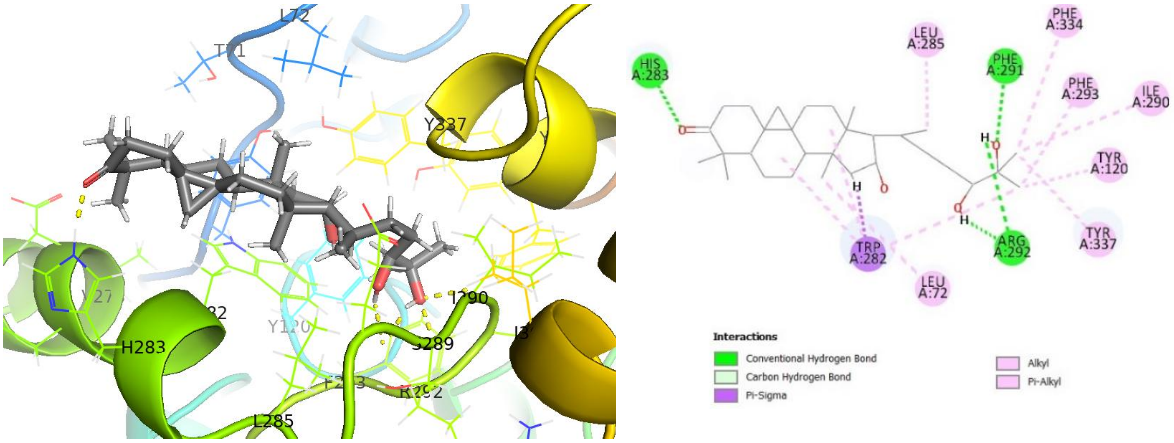

Figure S7. Molecular docking of compound **7** with acetylcholinesterase.

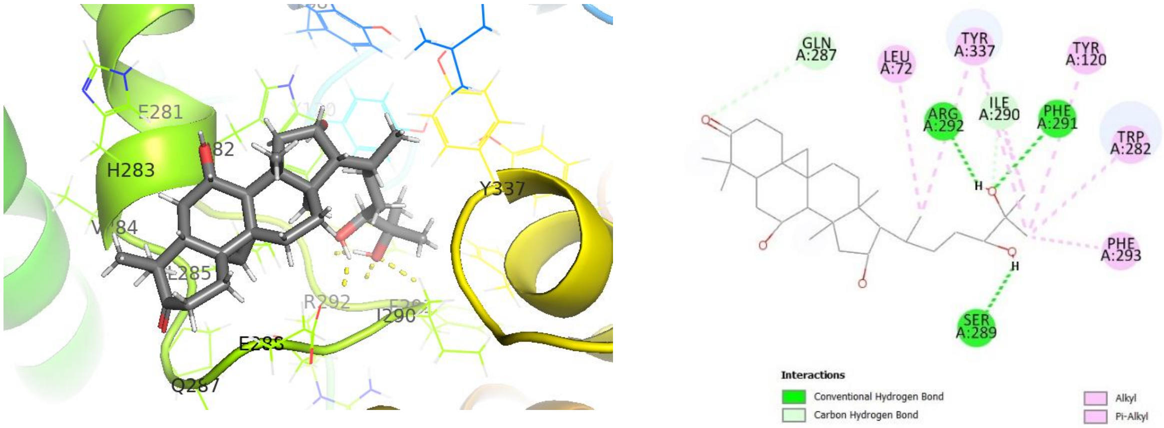

Figure S8. Molecular docking of compound 8 with acetylcholinesterase.

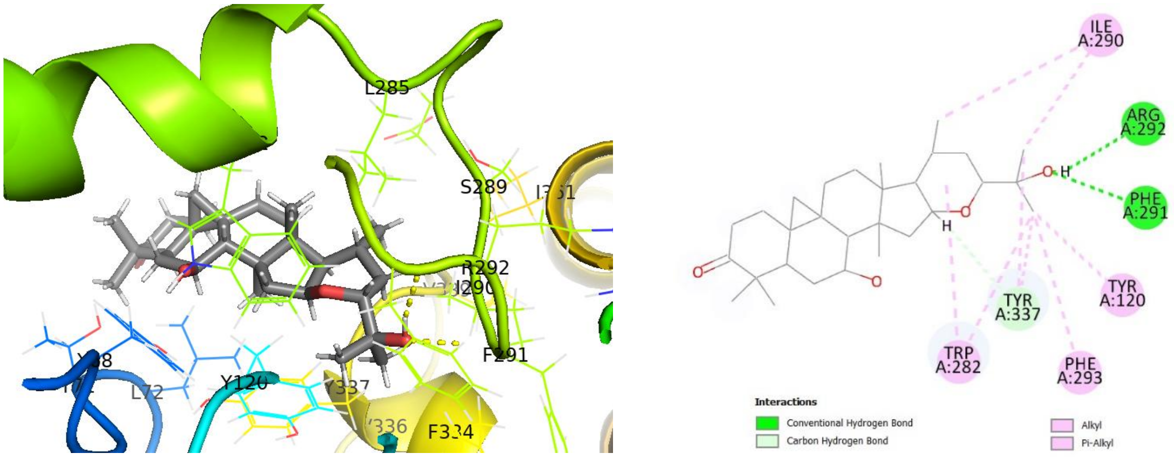

Figure S9. Molecular docking of compound **9** with acetylcholinesterase.

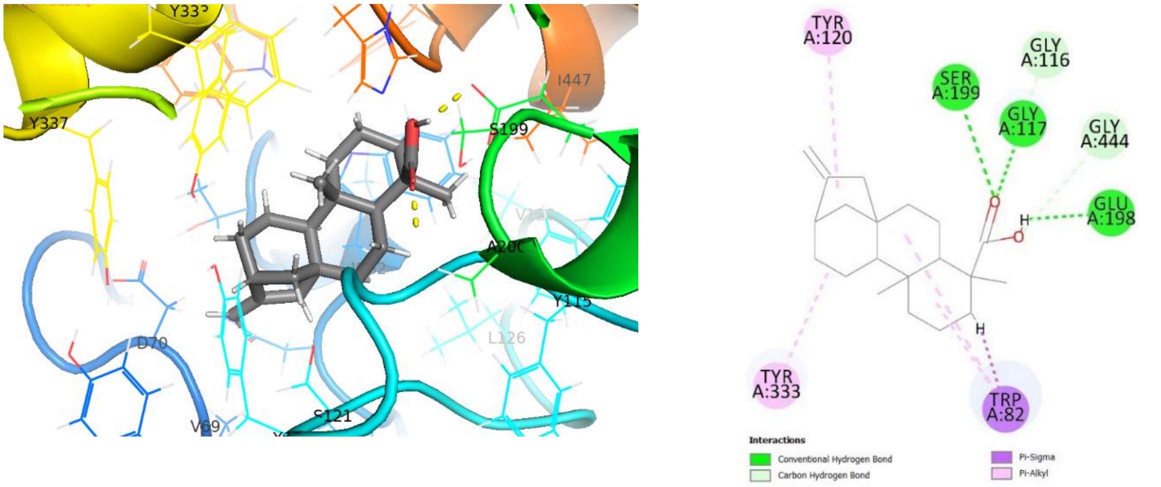

Figure S10. Molecular docking of compound **10** with acetylcholinesterase.

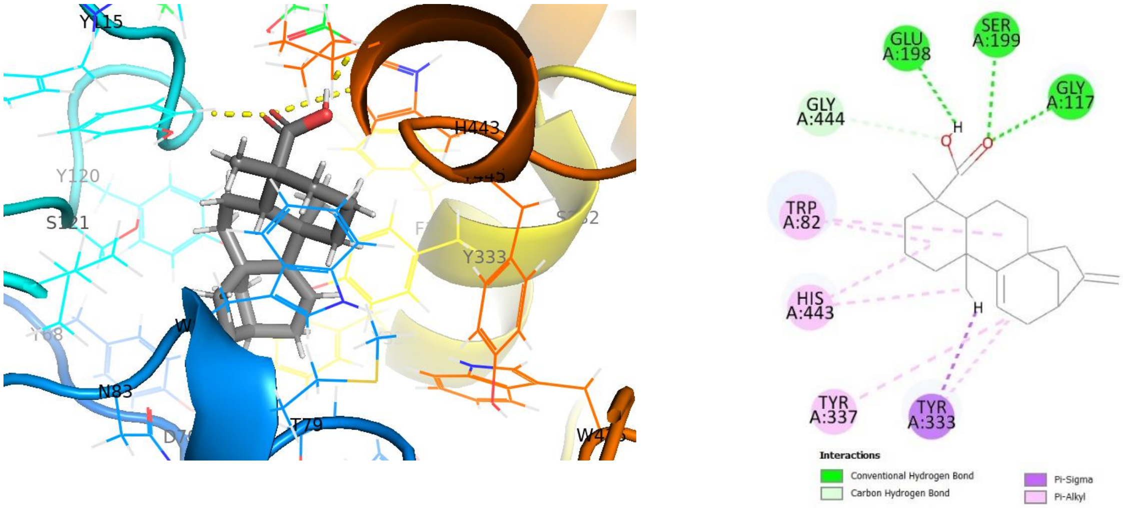

Figure S11. Molecular docking of compound **12** with acetylcholinesterase.

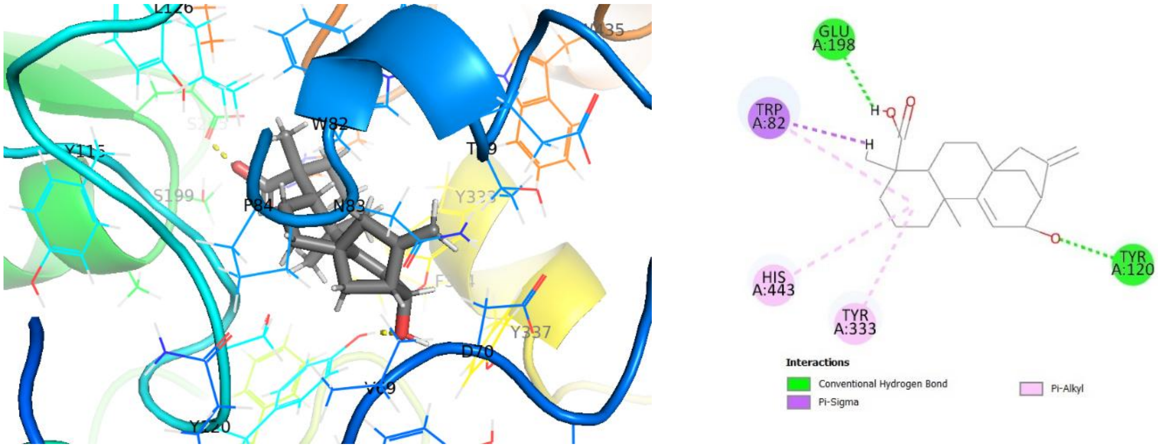

Figure S12. Molecular docking of compound **13** with acetylcholinesterase.

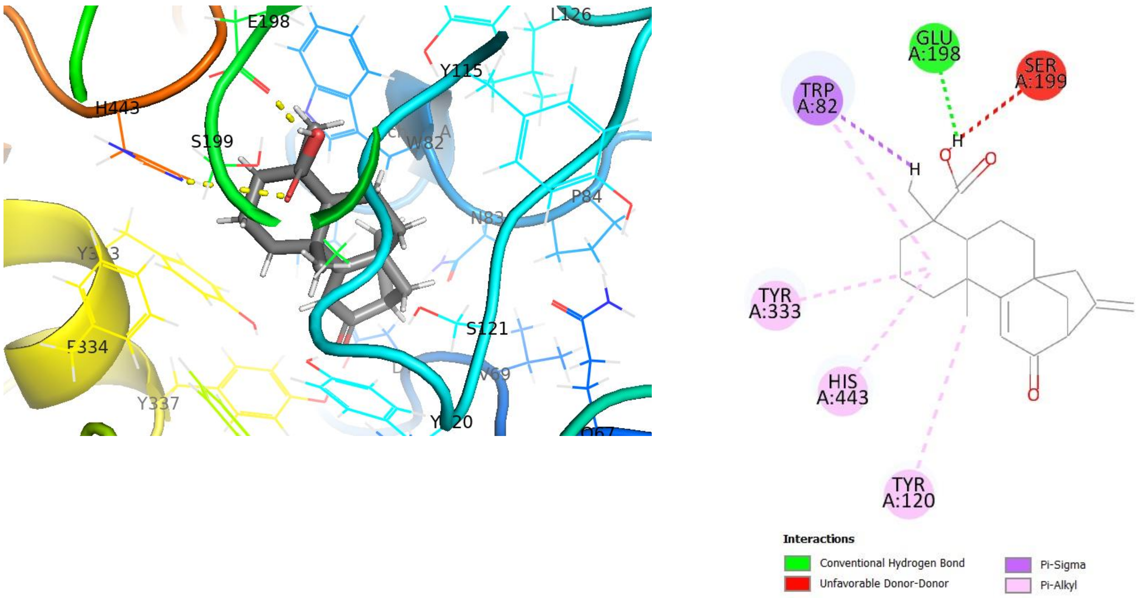

Figure S13. Molecular docking of compound **14** with acetylcholinesterase.

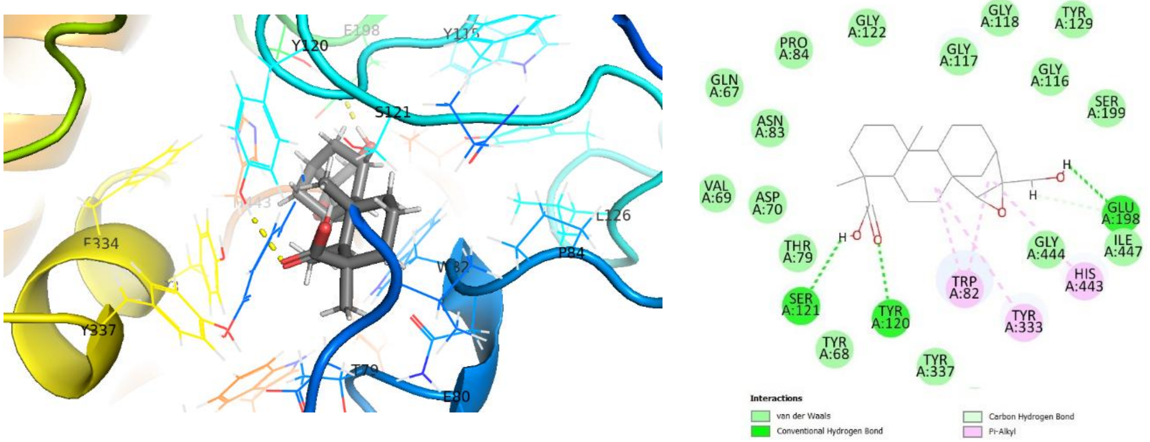

Figure S14. Molecular docking of compound **15** with acetylcholinesterase.

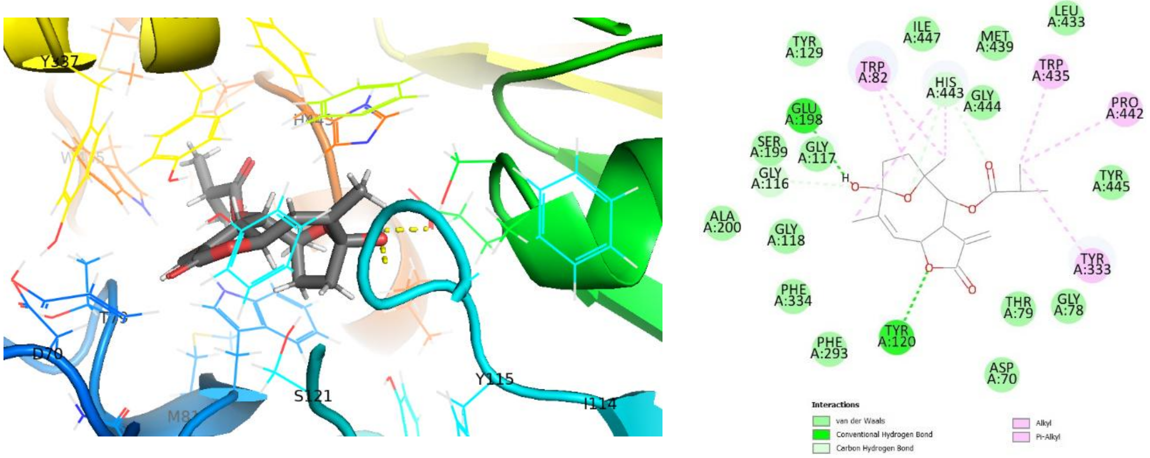

Figure S15. Molecular docking of compound **16** with acetylcholinesterase.

Table S1. Molecular Docking Analysis of Ligands Targeting Acetylcholinesterase for Potential Insecticidal Activity

Compound	Acetylcholinesterase Protein Residues Involved in Hydrogen Bonding with Ligand	Residues in AChE Involved in Non-polar Interactions with Ligand
**1**	P^{24}, Y^{101}	G^{23}, V^{25}, P^{100}, P^{102}, R^{103}, P^{104}, A^{105}, S^{106}, P^{107}, T^{108}, P^{109}, A^{135}, Q^{136}, G^{139}, R^{481}.
**2**	H^{283}, Y^{337}	Y^{68}, L^{72}, Y^{120}, W^{282}, V^{284}, L^{285}, Q^{287}, E^{288}, S^{289}, I^{290}, F^{291}, R^{292}, F^{293}, Y^{333}, F^{334}, G^{338}.
**3**	F^{291}	Y^{68}, Y^{120}, W^{282}, H^{283}, L^{285}, Q^{287}, E^{288}, S^{289}, I^{290}, R^{292}, F^{293}, Y^{333}, F^{334}, Y^{337}, G^{338}
**4**	F^{291}, Y^{337}	L^{72}, Y^{120}, W^{282}, H^{283}, L^{285}, Q^{287}, E^{288}, S^{289}, I^{290}, R^{292}, F^{293}, Y^{333}, F^{334}, G^{338}
**5**	S^{289}	Y^{68}, T^{71}, L^{72}, Q^{275}, V^{278}, D^{279}, H^{280}, W^{282}, H^{283}, L^{285}, E^{288}, I^{290}, F^{291}, R^{292}, F^{293}, F^{334}, Y^{337}, G^{338}.
**6**	-	Y^{68}, L^{72}, Y^{120}, W^{282}, L^{285}, E^{288}, S^{289}, I^{290}, F^{291}, R^{292}, F^{293}, Y^{333}, F^{334}, Y^{337}, G^{338}.
**7**	H^{283}, F^{291}, R^{292}-3 bonds	Y^{68}, T^{71}, L^{72}, Y^{120}, D^{279}, W^{282}, L^{285}, E^{288}, S^{289}, I^{290}, F^{293}, Y^{333}, F^{334}, Y^{337}, G^{338}
**8**	S^{289}, F^{291}, R^{292}-2 bonds	Y^{68}, L^{72}, Y^{120}, W^{282}, H^{283}, V^{284}, L^{285}, Q^{287}, E^{288}, I^{290}, F^{293}, Y^{333}, F^{334}, Y^{337}, G^{338}
**9**	F^{291}, R^{292}	Y^{68}, T^{71}, L^{72}, Y^{120}, W^{282}, L^{285}, E^{288}, S^{289}, I^{290}, F^{293}, Y^{333}, F^{334}, Y^{337}, G^{338}, I^{361}
**12**	G^{117}, E^{198}	Y^{68}, V^{69}, D^{70}, T^{79}, W^{82}, N^{83}, Y^{115}, G^{116}, G^{118}, Y^{120}, S^{121}, G^{122}, Y^{129}, S^{199}, Y^{333}, Y^{337}, H^{443}, G^{444}, Y^{445}, I^{447}
**13**	Y^{120}	Q^{67}, Y^{68}, V^{69}, D^{70}, T^{71}, G^{78}, T^{79}, E^{80}, W^{82}, N^{83}, P^{84}, G^{116}, G^{117}, G^{118}, S^{121}, G^{122}, E^{198}, S^{199}, Y^{333}, F^{334}, Y^{337}, L^{433}, W^{435}, M^{439}, P^{442}, H^{443}, G^{444}, Y^{445}, I^{447}.
**14**	G^{117}, E^{198}, H^{443}	D^{70}, T^{79}, E^{80}, W^{82}, N^{83}, G^{116}, G^{118}, Y^{120}, S^{121}, Y^{129} S^{199}, Y^{333}, F^{334}, Y^{337}, W^{435}, G^{444}, Y^{445}, I^{447}
**15**	G^{117}, Y^{120}, E^{198}	Y^{68}, V^{69}, D^{70}, T^{79}, E^{80}, W^{82}, N^{83}, G^{116}, G^{118}, S^{121}, G^{122}, Y^{129}, S^{199}, Y^{333}, F^{334}, Y^{337}, H^{443}, G^{444}, I^{447}
**16**	G^{117}, E^{198}, H^{443}	Y^{68}, V^{69}, D^{70}, T^{79}, E^{80}, W^{82}, N^{83}, P^{84}, G^{116}, G^{118}, Y^{120}, S^{121}, G^{122}, Y^{129}, S^{199}, Y^{333}, F^{334}, Y^{337}, G^{444}, I^{447}
